# Depressed β‐adrenergic inotropic responsiveness and intracellular calcium handling abnormalities in Duchenne Muscular Dystrophy patients’ induced pluripotent stem cell–derived cardiomyocytes

**DOI:** 10.1111/jcmm.16341

**Published:** 2021-02-22

**Authors:** Lucy N. Mekies, Danielle Regev, Binyamin Eisen, Jonatan Fernandez‐Gracia, Polina Baskin, Ronen Ben Jehuda, Rita Shulman, Irina Reiter, Raz Palty, Michael Arad, Eyal Gottlieb, Ofer Binah

**Affiliations:** ^1^ Department of Physiology Biophysics and Systems Biology Rappaport Faculty of Medicine Technion – Israel Institute of Technology Haifa Israel; ^2^ Department of Cell Biology and Cancer Science Rappaport Faculty of Medicine Technion – Israel Institute of Technology Haifa Israel; ^3^ Faculty of Biotechnology and Food Engineering Technion – Israel Institute of Technology Haifa Israel; ^4^ Department of Biochemistry Rappaport Faculty of Medicine Technion – Israel Institute of Technology Haifa Israel; ^5^ Leviev Heart Center Sheba Medical Center Ramat Gan Israel; ^6^ Sackler Faculty of Medicine Tel Aviv University Tel Aviv Israel

**Keywords:** [Ca^2+^]_i_ transients, contractions, duchenne muscular dystrophy, induced pluripotent stem cell–derived cardiomyocytes, RNA‐sequencing, SR Ca^2+^ stores, β‐adrenergic responsiveness

## Abstract

Duchenne muscular dystrophy (DMD), caused by mutations in the *dystrophin* gene, is an X‐linked disease affecting male and rarely adult heterozygous females, resulting in death by the late 20s to early 30s. Previous studies reported depressed left ventricular function in DMD patients which may result from deranged intracellular Ca^2+^‐handling. To decipher the mechanism(s) underlying the depressed LV function, we tested the hypothesis that iPSC‐CMs generated from DMD patients feature blunted positive inotropic response to β‐adrenergic stimulation. To test the hypothesis, [Ca^2+^]_i_ transients and contractions were recorded from healthy and DMD‐CMs. While in healthy CMs (HC) isoproterenol caused a prominent positive inotropic effect, DMD‐CMs displayed a blunted inotropic response. Next, we tested the functionality of the sarcoplasmic reticulum (SR) by measuring caffeine‐induced Ca^2+^ release. In contrast to HC, DMD‐CMs exhibited reduced caffeine‐induced Ca^2+^ signal amplitude and recovery time. In support of the depleted SR Ca^2+^ stores hypothesis, in DMD‐CMs the negative inotropic effects of ryanodine and cyclopiazonic acid were smaller than in HC. RNA‐seq analyses demonstrated that in DMD CMs the RNA‐expression levels of specific subunits of the L‐type calcium channel, the β1‐adrenergic receptor (ADRβ1) and adenylate cyclase were down‐regulated by 3.5‐, 2.8‐ and 3‐fold, respectively, which collectively contribute to the depressed β‐adrenergic responsiveness.

## 1 INTRODUCTION

Duchenne muscular dystrophy (DMD), the most common of 9 types of muscular dystrophy, is an X‐linked disease affecting boys and teenagers and rarely adult heterozygous females. The incidence in male newborns is 1:3500, and the prevalence is 6:100 000 in the male population.^1^ The disease is caused by mutations in the *dystrophin* gene encoding the dystrophin protein and constitutes the most severe childhood form of the broader family of muscular dystrophies.^2^, ^3^, ^4^ Dystrophin is a key structural/functional protein providing strength and stability to the contracting muscle and is essential for maintaining healthy muscle function; its lack leads to cell damage, impaired Ca^2+^ homeostasis, elevated oxidative stress and reduced energy production in muscle cells. Consequently, loss of sarcolemmal dystrophin and dystrophin‐glycoprotein complex (DGC) promotes muscle fibre damage during muscle contraction.^5^ Symptoms onset usually occurs between ages 3 and 5, and includes progressive muscle weakness and wasting.^1^, ^2^ By the late teens, the heart and respiratory muscles are also affected, ultimately leading to death due to respiratory and/or cardiac failure.^6^, ^7^

Dilated cardiomyopathy (DCM), which is a key pathological feature in DMD patients, affects nearly all patients and is a major cause of morbidity and mortality.^4^, ^7^, ^8^ Accordingly, many studies reported depressed left ventricular contractile function in DMD patients^9^, ^10^, ^11^, ^12^, ^13^, ^14^, ^15^ which may result from impaired β‐adrenergic signalling cascade and/or intracellular Ca^2+^‐handling. The latter is supported by: (a) our recent study in DMD induced pluripotent stem cell–derived cardiomyocytes (iPSC‐CMs) demonstrating prominent arrhythmias in the form of delayed afterdepolarizations (DADs) known to result from Ca^2+^‐overload^16^; (b) studies in *mdx* mice cardiomyocytes showing ‘leaky’ Ryanodine receptor (RyR) and abnormal Ca^2+^ handling.^17^, ^18^, ^19^ To decipher the mechanism(s) underlying the depressed ventricular function in DMD patients, we tested the hypothesis that DMD patients' iPSC‐CMs feature blunted positive inotropic response to β‐adrenergic stimulation. Our major findings are: (a) the inotropic responses to isoproterenol and elevated [Ca^2+^]_o_ are blunted in DMD cardiomyocytes; (b) defective β‐adrenergic receptor cascade is not responsible for the blunted inotropic response in DMD cardiomyocytes; (c) sarcoplasmic reticulum (SR) Ca^2+^‐handling machinery is altered in DMD cardiomyocytes; (d) depressed SR Ca^2+^ release in DMD cardiomyocytes is likely to underlie the attenuated positive inotropic response; and (e) RNA‐expression levels of specific subunits of the L‐type calcium channel, the β1‐adrenergic receptor (ADRβ1) and adenylate cyclase are down‐regulated in DMD cardiomyocytes by 3.5‐, 2.8‐ and 3‐fold, respectively, which may collectively contribute to the depressed β‐adrenergic responsiveness. These novel findings provide an explanation for the depressed ventricular function in DMD patients and thus may help to introduce therapeutic support for the patients.

## 2 METHODS

### 2.1 Tissue collection from DMD patients

Dermal biopsies were obtained from a 50‐year‐old DMD female manifesting carrier with a deletion of exons 8‐12 (ex.8_12del) and a 32‐year‐old DMD male patient carrying a substitution of cytosine to thymine (c.5899C>T) constituting a premature stop codon. The donors signed a consent form according to approval #7603‐09‐SMC by the Helsinki Committee for Experiments on Human Subjects at Sheba Medical Center, Ramat Gan, Israel. iPSCs generation and characterization, karyotype analysis, teratoma formation, genotyping and pluripotency evaluation by immunofluorescence staining and flow cytometry were previously described.^16^ Differentiation into cardiomyocytes was performed according to the directed differentiation by modulating Wnt/β‐catenin signalling.^20^

### 2.2 Measurements of intracellular Ca^2+^ transients, contractions and action potentials

All methods are detailed in Supplementary Material. In brief, intracellular Ca^2+^ ([Ca^2+^]_i_) transients and contractions were measured from iPSC‐CMs composing embryoid bodies (EBs), using the IonOptix Calcium and Contractility system (Westwood, MA, USA), as previously described.^21^, ^22^ Transmembrane action potentials were recorded by means of the whole cell patch clamp.^16^, ^23^

### 2.3 Western blot

See details in Supplementary Material.

### 2.4 RNA extraction and RNA‐seq analysis

RNA was extracted from control and DMD cardiomyocytes using the ‘ReliaPrep™ RNA Cell Miniprep System Kit’ (Promega, Fitchburg, WI, USA). RNA‐seq libraries were produced and the RNA‐seq data were analysed. See details in Supplementary Material. The raw RNA‐Seq data were deposited and released in the SRA database, with the BioProject accession number PRJNA688142.

### 2.5 Statistical analysis

Results are presented as mean ± SEM. See details in Supplementary Material.

## 3 RESULTS

### 3.1 DMD iPSC‐CMs exhibit blunted β‐adrenergic inotropic responsiveness

**FIGURE 1:**
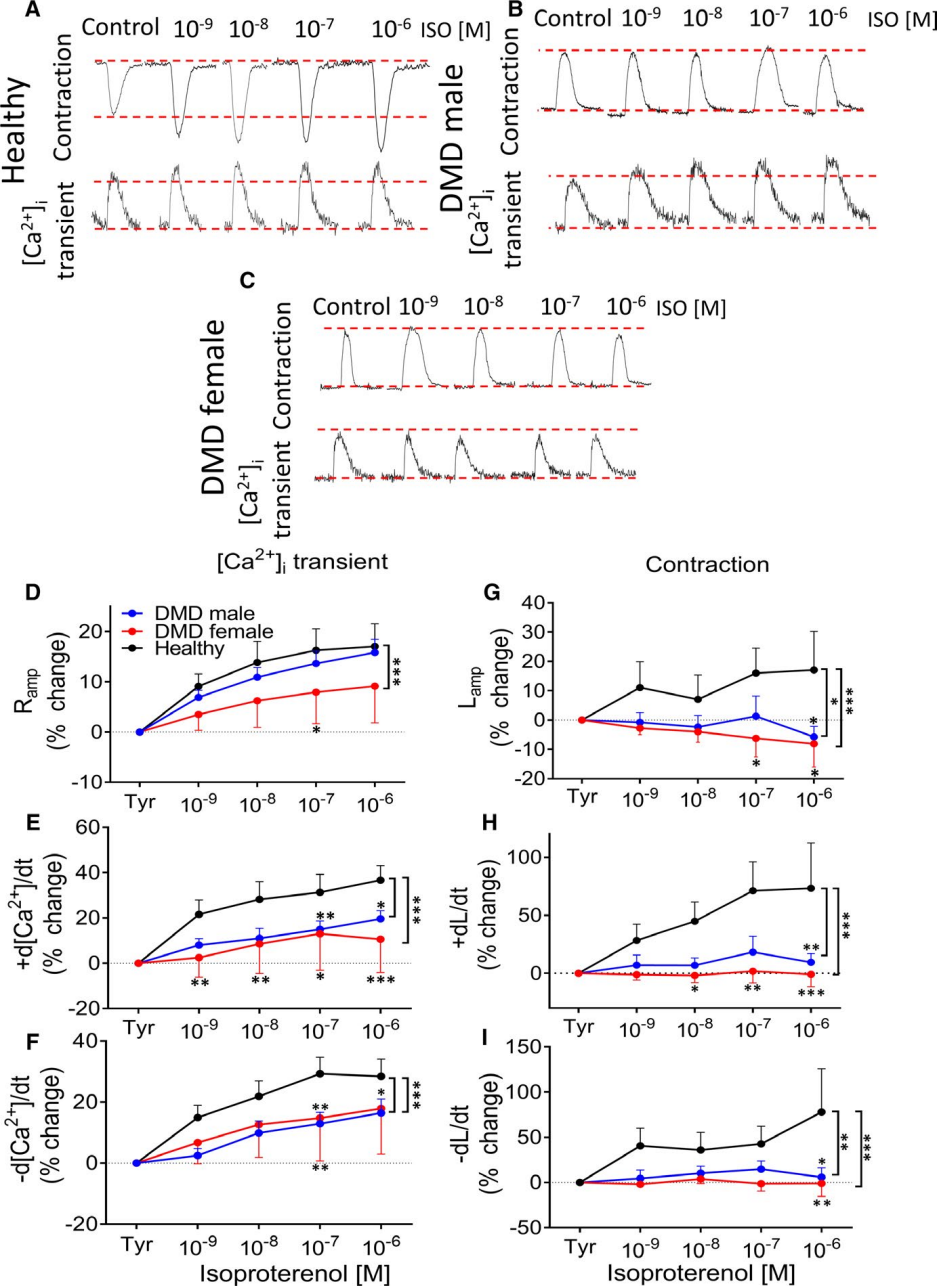
The effects of increased isoproterenol concentrations on the [Ca^2+^]_i_ transients and contractions in healthy, DMD adult male and adult female iPSC‐CMs. (A‐C) Representative [Ca^2+^]_i_ transients and contractions from healthy, adult male and adult female iPSC‐CMs in the absence (Tyrode's, Tyr) and presence of isoproterenol. (D) [Ca^2+^]_i_ transient amplitude (Ramp). (E) Maximal rate of [Ca^2+^]_i_ rise (+d[Ca^2+^]/dt). (F) Maximal rate of [Ca^2+^]_i_ decay (−d[Ca^2+^]/dt). (G) Maximal amplitude (Lamp). (H) Maximal contraction rate (+dL/dt).(I) Maximal relaxation rate (−dL/dt). iPSC‐CMs were stimulated at a frequency 20% higher than the spontaneous firing rate. The effect of isoproterenol on the [Ca^2+^]_i_ transient parameters of adult male (n = 12), adult female (n = 20) and healthy (n = 14)cardiomyocytes was expressed as per cent change from control (Tyrode's, Tyr). The effect of isoproterenol on the contraction parameters of adult male (n = 11), adult female (n = 14) and healthy (n = 7) cardiomyocytes was expressed as per cent change from control (Tyrode's, Tyr). Tyr: Tyrode's solution; *P < 0.05, **P < 0.01, ***P < 0.001 (vs control, Tyrode's). Two‐way ANOVA test was performed followed by Holm‐Sidak *post* *hoc* test

All control experiments were performed on healthy male and female (clones fse5m and 24.5) cardiomyocytes. The first step was to determine whether the two healthy clones have similar [Ca^2+^]_i_‐handling and contractile machineries. As seen Figure S1, the [Ca^2+^]_i_ transient and contraction parameters are similar in the two healthy clones (fse5m and 24.5), and therefore, one lumped healthy (control) group was used for comparison with adult male and adult female DMD cardiomyocytes. In support of our hypothesis, adult male and adult female DMD cardiomyocytes displayed a blunted positive inotropic response to isoproterenol compared to healthy cells (Figure 1). Specifically, except for [Ca^2+^]_i_ transient amplitude in adult male, all other [Ca^2+^]_i_ transient parameters in adult male and adult female were augmented to a smaller extent by isoproterenol, compared to healthy cells. A similar blunted response in adult male and adult female cardiomyocytes was observed respecting the contraction parameters; contraction amplitude (L_amp_), maximal rate of contraction (+dL/dt) and maximal rate of relaxation (−dL/dt) (Figure 1G‐I). The extrapolation of these results to DMD patients sheds light on the mechanisms underlying their depressed ventricular function.

### 3.2 Are the[Ca^2+^]_i_‐handling and contractile machineries depressed in DMD cardiomyocytes?

To determine whether the blunted β‐adrenergic inotropic response in DMD cardiomyocyte is due to depressed [Ca^2+^]_i_‐handling and contractile machineries we compared [Ca^2+^]_i_ transient and contraction parameters in healthy, adult male and adult female iPSC‐CMs. As illustrated in Figure S2, [Ca^2+^]_i_ transient amplitude and all the contraction parameters were comparable in DMD and healthy cardiomyocytes. The exceptions were: (a) maximal rate of [Ca^2+^]_i_ rise in adult male was smaller than in healthy cardiomyocytes (Figure S2B); (b) maximal rate of [Ca^2+^]_i_ rise in adult female was larger than healthy and adult male cardiomyocytes (Figure S2B); (c) maximal rate of [Ca^2+^]_i_ decay in adult male was larger than healthy cardiomyocytes (Figure S2C); (d) maximal rate of [Ca^2+^]_i_ decay in adult female was smaller than adult male cardiomyocytes (Figure S2C). Collectively, these findings show that the [Ca^2+^]_i_ transients and contractions are not impaired in DMD cardiomyocytes, indicating that the blunted β‐adrenergic inotropic response did not result from depressed [Ca^2+^]_i_‐handling and contractile machineries.

### 3.3 Is the β‐adrenergic signalling cascade impaired in DMD cardiomyocytes?

**FIGURE 2:**
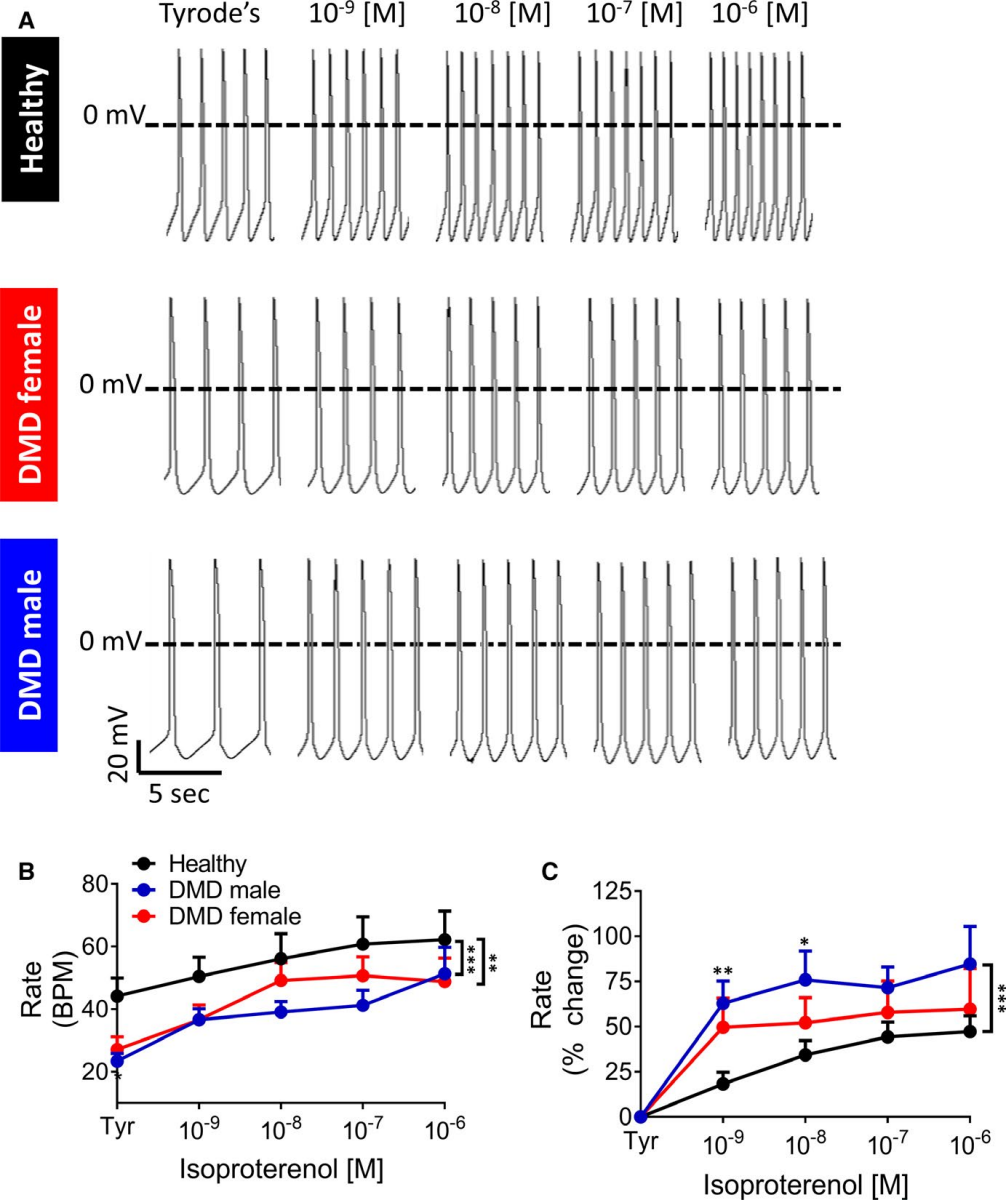
Changes in the spontaneous firing rate of iPSC‐CMs in response to β‐adrenergic stimulation by isoproterenol. (A) Representative spontaneuosly firing action potential recordings in the absence (Tyrode's, Tyr) and presence of isoproterenol (10^−9^‐10^−6^ mol/L), from healthy (top), DMD adult female (middle), and DMD adult male (bottom) iPSC‐CMs. (B, C) Changes in the spontaneous firing rate in response to isoproterenol (10^−9^‐10^−6^ mol/L). (B)Rate (Beats Per Minute, BPM); (C) per cent change in Rate compared to control (Tyrode's, Tyr). Healthy, n = 14; adult female, n = 11; adult male, n = 11. Two‐way ANOVA followed by Holm‐Sidak *post* *hoc* test. *P < 0.05, **P < 0.01, ***P < 0.001

To determine whether the blunted inotropic response was caused by impaired β‐adrenergic signalling cascade in DMD cardiomyocytes, we measured the positive chronotropic response to isoproterenol. As we previously reported^16^ and shown here as well (Figure 2A,B), DMD cardiomyocytes exhibit lower (*P* < 0.001 and *P* < 0.01 in adult male and adult female, respectively) spontaneous firing rate compared to healthy cardiomyocytes. In support of the functionality of the β‐adrenergic signalling cascade, adult male and adult female cardiomyocytes presented a healthy‐like positive chronotropic response to isoproterenol. Further, adult male cardiomyocytes exhibited a larger chronotropic response than healthy cells (Figure 2C), which is in agreement with the increased heart rate in DMD patients.^24^ From these experiments we concluded that an impaired β‐adrenergic cascade is not responsible for the blunted positive inotropic response in DMD cardiomyocytes.

### 3.4 Intracellular Ca^2+^ handling machinery is altered in DMD cardiomyocytes

Since we excluded the possibility that the blunted inotropic response is due to depressed [Ca^2+^]_i_‐handling and contractile machineries, we tested the hypothesis that impaired downstream element(s) mediating positive inotropic interventions is depressed in DMD cardiomyocytes.

#### 3.4.1 The ionotropic effect of elevated [Ca^2+^]_o_

**FIGURE 3:**
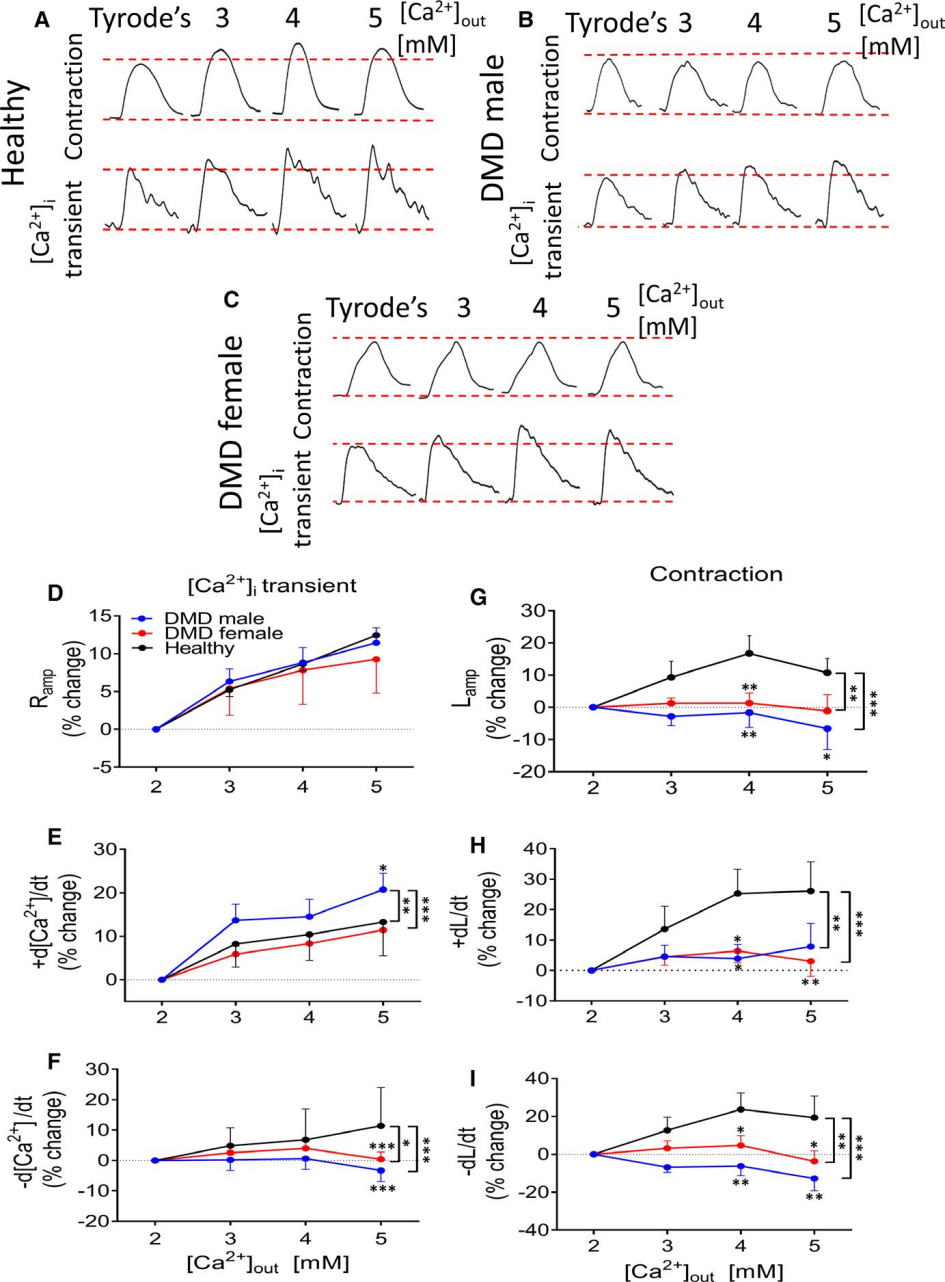
The effects of different concentrations of [Ca^2+^]_o_ on the [Ca^2+^]_i_ transients and contractions in healthy, DMD adult male and DMD adult female iPSC‐CMs. (A‐C) Representative [Ca^2+^]_i_ transients and contractions from healthy, adult male and adult female iPSC‐CMs in the presence of different concentrations of [Ca^2+^]_o_. (D) [Ca^2+^]_i_ transient amplitude (Ramp). (E) Maximal rate of [Ca^2+^]_i_ rise (+d[Ca^2+^]/dt). (F) Maximal rate of [Ca^2+^]_i_ decay (−d[Ca^2+^]/dt). (G) Maximal amplitude (Lamp). (H) Maximal contraction rate (+dL/dt). (I) Maximal relaxation rate (−dL/dt). iPSC‐CMs were stimulated at a frequency 20% higher than the spontaneous firing rate. The effect of different concentrations of [Ca^2+^]_o_on the [Ca^2+^]_i_ transient parameters of adult male (n=11), adult female (n=15) and healthy, n = 22) cardiomyocytes was expressed as per cent change of Control (2 mmol/L [Ca^2+^]_o_ The effect of different concentrations of [Ca^2+^]_o_ on contraction parameters of adult male (n=10), adult female (n=17) and healthy (n=17) cardiomyocytes was expressed as per cent change of Control (2 mmol/L [Ca^2+^]_o_. *P < 0.05, **P < 0.01, ***P < 0.001 (vs Control, 2 mmol/L [Ca2+]_o_). Two‐way ANOVA test was performed followed by Holm‐Sidak *post* *hoc* test

Here, we tested the inotropic response to a non‐receptor–mediated positive inotropic intervention, elevated [Ca^2+^]_o_ (2, 3, 4 and 5 mmol/L), which operates by augmenting the L‐type Ca^2+^ current (I_Ca,L_), which in turn increases SR Ca^2+^ release and contractile force.^25^ As seen in Figure 3A, while in healthy cardiomyocytes, elevating [Ca^2+^]_o_ caused a positive inotropic effect, DMD cardiomyocytes were hardly affected (Figure 3B‐C). Specifically, except for [Ca^2+^]_i_ transient amplitude, the response of all other [Ca^2+^]_i_ transient and contraction parameters to elevated [Ca^2+^]_o_ was blunted in DMD compared to healthy cardiomyocytes (Figure 3D‐I).

#### 3.4.2 SR Ca^2+^ handling

Because the inotropic response to isoproterenol and elevated [Ca^2+^]_o_ was blunted in DMD cardiomyocytes, we hypothesized that this was due to a downstream common element—depleted SR Ca^2+^ stores. This hypothesis was tested by investigating: (a) caffeine‐induced RyR‐mediated SR Ca^2+^ release; (b) the inotropic effects of ryanodine and cyclopiazonic acid (CPA), both interfering with SR Ca^2+^‐handling.^26^, ^27^

#### 3.4.3 Caffeine‐induced RyR‐mediated SR Ca^2+^ release

**FIGURE 4:**
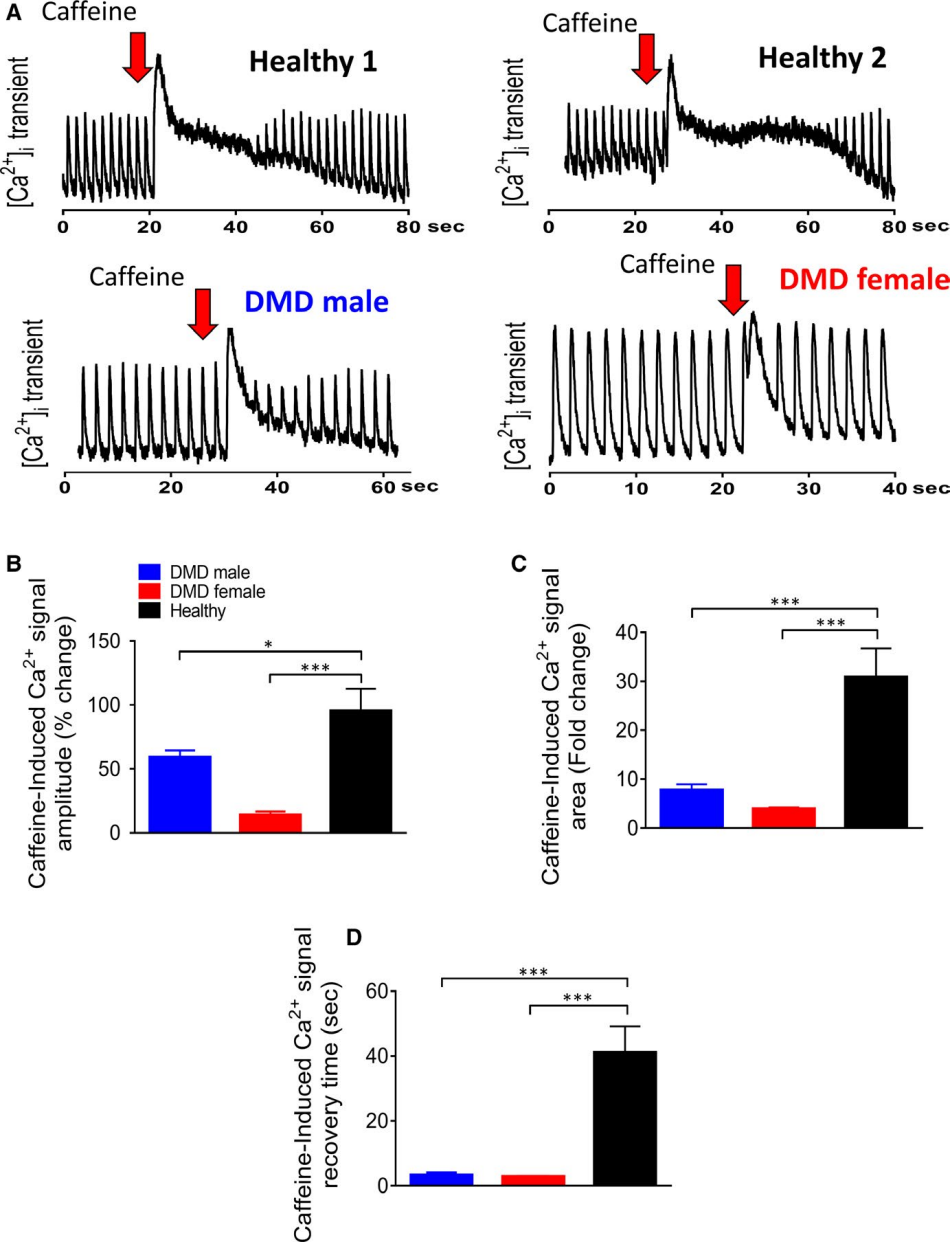
The effects of caffeine (10 mmol/L) on healthy, DMD adult male and DMD adult female iPSC‐CMs. (A) Representative [Ca^2+^]_i_ transients from healthy, DMD adult male and DMD adult female iPSC‐CMs demonstrate the effect of caffeine. (B) Per cent change in caffeine‐induced [Ca^2+^]_i_ signal amplitude compared to the pre‐caffeine amplitude; (C) Per cent change in area of the caffeine‐induced [Ca^2+^ ]_i_ signal compared to the pre‐caffeine area; (D) The mean recovery time, calculated as the time from the peak of caffeine‐induced [Ca^2+^]_i_ rise to the first measurable [Ca^2+^]_i_ transient. Adult male (n = 16) iPSC‐CMs; adult female (n = 8) iPSC‐CMs; healthy (n = 16) iPSC‐CMs.          *P < 0.05, ***P < 0.001. Asterisks above bars connecting columns represent significant difference between groups. One‐way ANOVA test was followed by Holm‐Sidak *Post* *hoc* test

RyR‐mediated SR Ca^2+^ release was measured by a brief application of caffeine (10 mmol/L), a RyR2 opener.^28^ As illustrated in Figure 4, DMD and healthy cardiomyocytes differed in their response to caffeine. As we previously reported,^21^, ^22^, ^29^ in healthy cardiomyocytes caffeine caused an abrupt increase in [Ca^2+^]_i_ associated with contraction cessation, followed by a decline in [Ca^2+^]_i_ along with resumption of contractions after ~25 seconds (Figure 4A). In contrast, the response of adult male and adult female cardiomyocytes was shorter and smaller (Figure 4B‐D). The caffeine response was quantified by calculating 3 parameters: (a) recovery time—the time from the peak of caffeine‐induced [Ca^2+^]_i_ rise to the first [Ca^2+^]_i_ transient; (b) the per cent change in caffeine‐induced [Ca^2+^]_i_ response amplitude, compared to pre‐caffeine amplitude; and (c) the per cent change in caffeine‐induced [Ca^2+^]_i_ response area, compared to the pre‐caffeine value. In summary, in both adult male and adult female compared to healthy cardiomyocytes: (a) the area and amplitude were smaller (*P < 0.05, ***P < 0.001; Figure 4B,C). (b) the recovery time was shorter (***P < 0.001; Figure 4D). In summary, depressed SR Ca^2+^ release in DMD cardiomyocytes is likely to underlie the attenuated positive inotropic responsiveness to isoproterenol and elevated [Ca^2+^]_o_.

#### 3.4.4 The negative inotropic effects of ryanodine and cyclopiazonic acid (CPA) on DMD iPSC‐CMs

**FIGURE 5:**
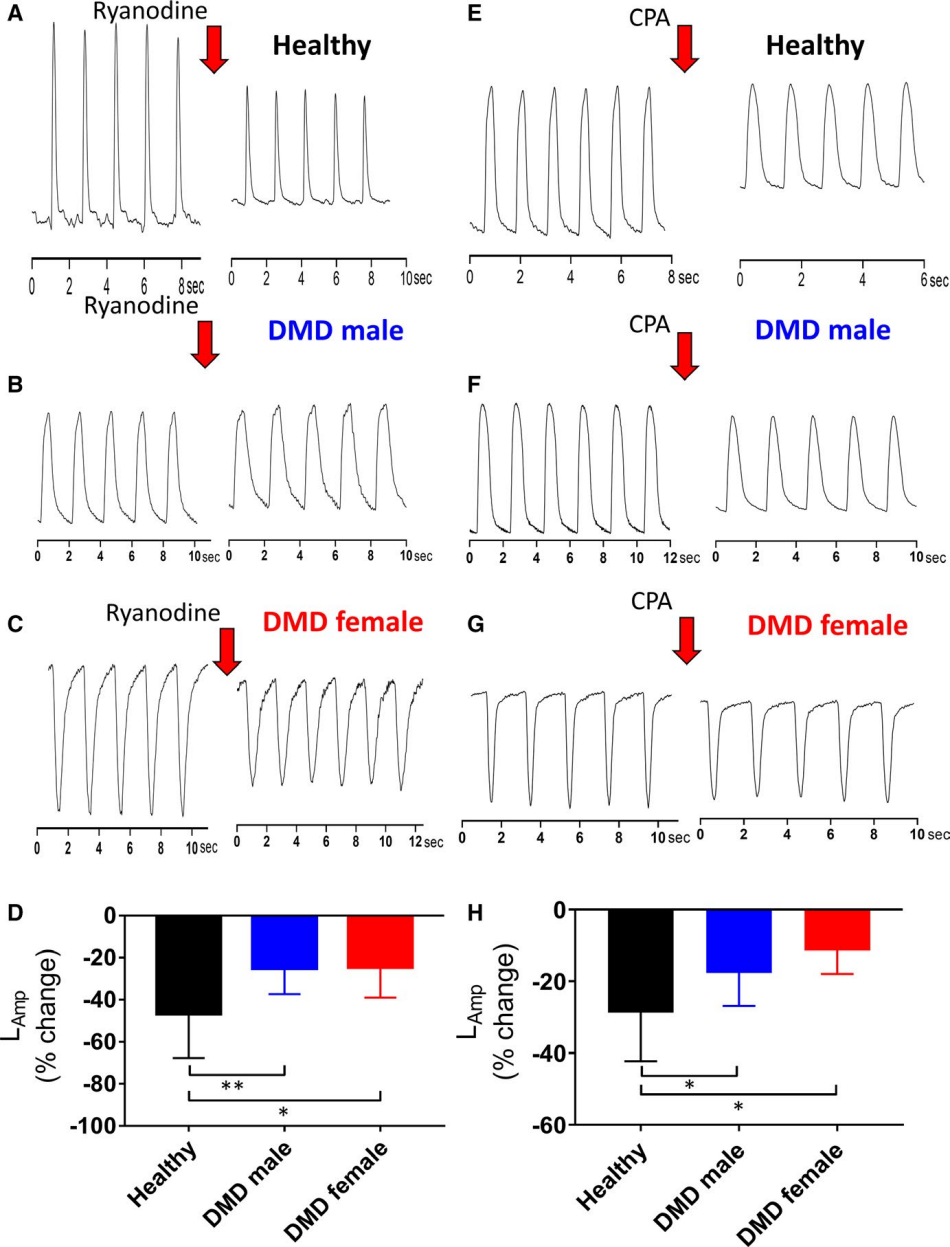
The effects of ryanodine (30 mmol/L) on healthy (A), DMD adult male (B) and DMD adult female iPSC‐CMs (C). iPSC‐CMs were stimulated at a frequency 20% higher than the spontaneous rate. (D) The effect of ryanodine on contraction amplitude (L_Amp_)of adult male (n = 11), adult female (n = 5) and healthy (n = 21) iPSC‐CMs was expressed as per cent change from Control (no drug). Tyr, Tyrode's solution; *P < 0.05, **P < 0.01, ***P < 0.001 (vs control, no drug). One‐way ANOVA test was performed followed by *Post* *hoc* Holm‐Sidak test. The effects of cyclopiazonic acid (CPA) (30 mmol/L) on healthy (E), adult male (F) and adult female iPSC‐CMs (G). **i**PSC‐CMs were stimulated at a frequency 20% higher than the spontaneous rate. (H), The effect of cyclopiazonic acid (30 mmol/L) on the contraction amplitude (LAmp) of adult male (n = 17), adult female (n = 6) and healthy (n = 14) iPSC‐CMs was expressed as per cent change from Control (no drug). *P < 0.05, **P < 0.01, ***P < 0.001 (vs healthy). One‐way ANOVA test was performed followed by Holm‐Sidak *Post* *hoc* test

If SR Ca^2+^ stores are depleted in DMD cardiomyocytes, ryanodine (30 μmol/L, Ref. 30) is expected to have a smaller inhibitory effect than in healthy cells. Indeed, as depicted by the representative contraction recordings and the summary, the contraction amplitude was decreased to a lesser (*P* < 0.01, *P* < 0.05) extent in adult male and adult female respectively, than in healthy cardiomyocytes (Figure 5A‐D). To further support the DMD depleted SR Ca^2+^ stores hypothesis, we investigated the negative inotropic effect of CPA, a SERCA inhibitor.^27^ Like ryanodine, the contraction amplitude was less reduced in adult male and adult female than in healthy cardiomyocytes (Figure 5E‐H).

### 3.5 The molecular mechanisms underlying the blunted positive inotropic response in DMD cardiomyocytes

**FIGURE 6:**
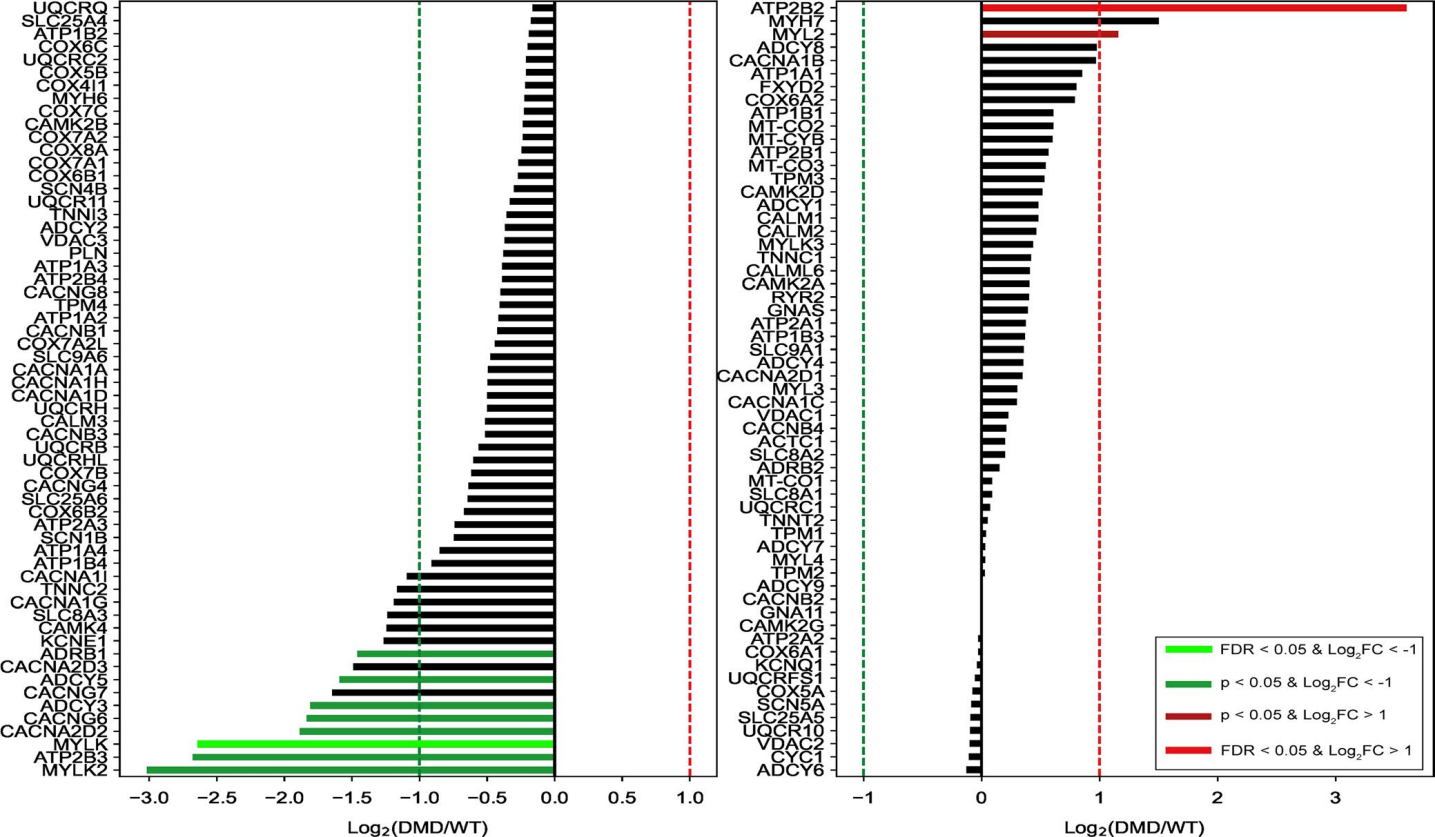
Waterfall barplot of gene expression changes between DMD and healthy iPSC‐CMs. Colours represent the level of significance in the down‐regulated (green) and up‐regulated (red) DEGs (differently expressed genes). Dark colours indicate significance at the nominal *P*‐value level, and bright colours indicate significance at the FDR‐corrected *P*‐value level, alpha = 0.05. X‐axis exhibits fold change in gene expression (Log2(DMD/WT))

**FIGURE 7:**
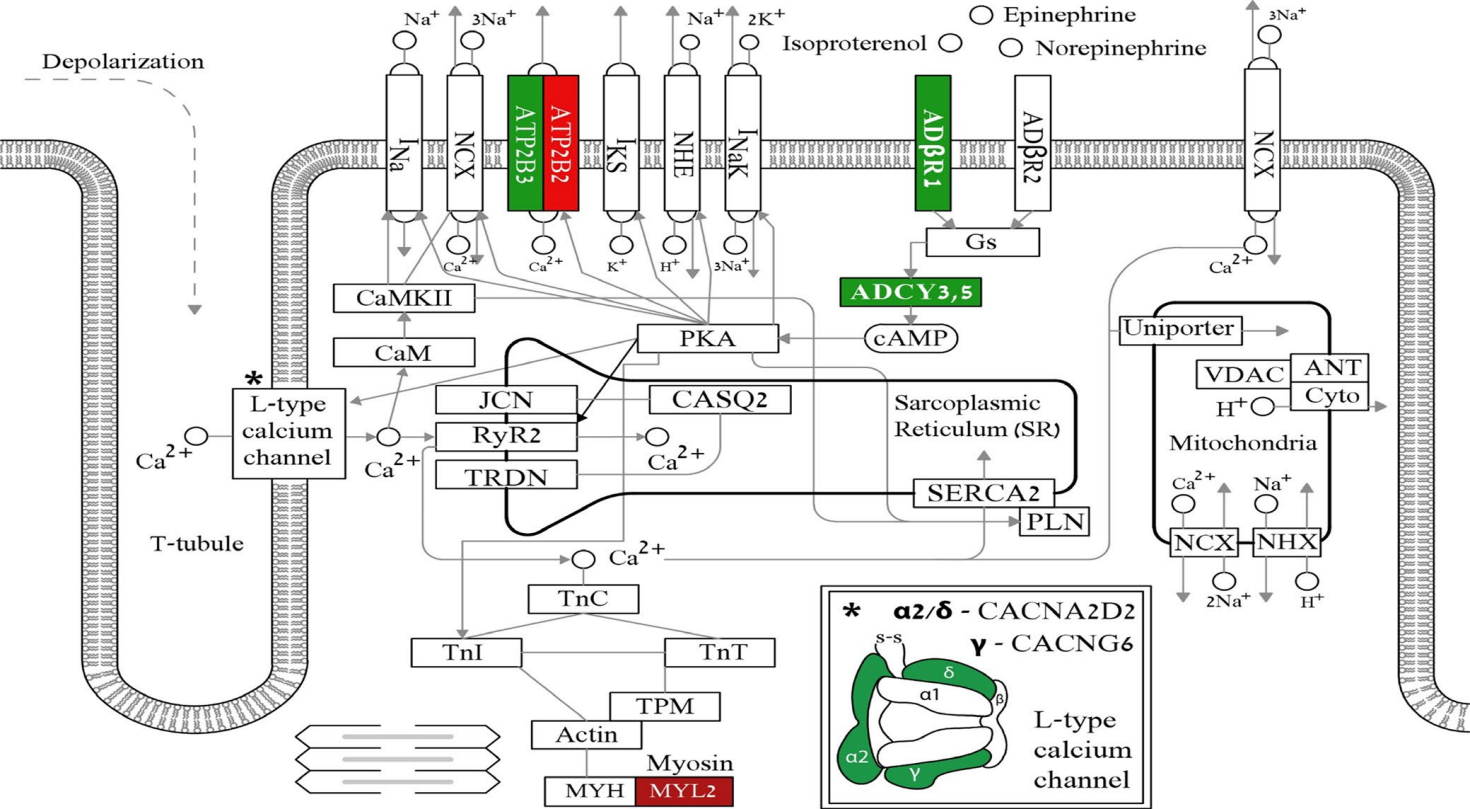
Integrated map consisting of key components of contraction, calcium signalling and adrenergic signalling pathways in cardiomyocytes. Colours are concordant with the waterfall and represent the level of significance in the down‐regulated (green) and up‐regulated (red) DEGs (differently expressed genes). Dark colours indicate significance at the nominal *P*‐value level, and bright colours indicate significance at the FDR‐corrected (false discovery rate) *P*‐value level, *P* ˂ 0.05

To decipher the molecular mechanisms underlying the blunted positive inotropic response in DMD cardiomyocytes, we carried out a comprehensive RNA‐seq analysis, comparing healthy and DMD cardiomyocytes. Altogether, the analysis (see Methods) detected 19 389 genes, of which we focused on 119 genes that were included in the KEGG (Kyoto Encyclopedia of Genes and Genomes) pathways for (cardiac) intracellular calcium signalling, contraction and adrenergic signalling. Briefly, KEGG is a database resource for understanding high‐level functions of biological systems, such as the cell, the organism and the ecosystem, from molecular‐level information, especially large‐scale molecular data sets generated by genome sequencing and other high‐throughput experimental technologies. Accordingly, all 119 genes included in the 3 KEGG pathways are illustrated in the combined waterfall plot (Figure 6). The differently expressed genes (DEGs) are aligned according to the degree of change of the expression level, measured by fold change in DMD cardiomyocytes compared to healthy control. For the sake of clarity, the waterfall was split into a left panel and a right panel, including down‐regulated and up‐regulated genes, respectively. Significant changes in expression levels are indicated by colour. Bright colours indicate significant changes at the False Discovery Rate (FDR)‐corrected *P*‐value, and milder colours indicate significance according to nominal *P*‐value. In an attempt to explain the functional findings described above, based on the KEGG and the corresponding waterfall data, we generated a map (Figure 7) which depicts the major elements of the intracellular calcium signalling, contraction and adrenergic signalling, albeit the extrapolation from transcriptomic data to function is not straightforward. Notably, there are many more genes in the waterfall plot than boxes in the map, since many of the boxes refer to multimeric complexes or to a class of receptors, including some referencing to over a dozen different genes, such as DHPR, PP2A and GPCR. The first functional finding supported by the RNA‐seq data is that the [Ca^2+^]_i_‐handling and contractile machineries properties are comparable in healthy and DMD cardiomyocytes (Figure S2). Specifically, key elements of these machinaries —RyR2 (type 2 Ryanodine receptor), SERCA2 (Sarcoplasmic/Endoplasmic Reticulum type 2), calsequestrin (CASQ2), phospholamban (PLN), troponins and actin—are all similar in both groups. As depicted in Figure 7, several alternations in gene expression were detected in discrete components constituting the myosin light chain complex. Specifically, whereas myosin light chain kinase (MYLK) and MYLK2 are down‐regulated by ~6‐fold in DMD cardiomyocytes, myosin light chain 2 (MYL2) is up‐regulated by 2‐fold. Further, we found down‐regulation by 3.5‐fold of genes that encode proteins of specific subunits of the L‐type calcium channel, CACNA2D2 and CACNG6, encoding for alpha‐2/delta subunit and gamma subunit, respectively. Finally, two genes that encode the plasma membrane Ca^2+^‐pump are differentially regulated in DMD cardiomyocytes; ATP2B3 is down‐regulated while ATP2B2 is up‐regulated. The next key functional finding for which we searched for support in the RNA‐seq data is the depressed β‐adrenergic inotropic response in DMD cardiomyocytes. Indeed, the expression of the β1‐adrenergic receptor (ADRβ1) which predominantly mediates the positive inotropic effect isoproterenol in the heart^31^, ^32^, ^33^; is down‐regulated in DMD cardiomyocytes by 2.8‐fold, compared to healthy cells. In contrast, the expression of β2‐adrenergic receptor (ADRβ2) which accounts for ~20% of all cardiac β‐adrenoreceptors,^33^ and whose activation increases contractile force and heart rate, is not altered in DMD cardiomyocytes. A major finding clearly contributing to the depressed β‐adrenergic inotropic response, is the >3‐fold decrease in the expression levels of the key enzyme adenylate cyclase (AC) isoforms III and V (ADCY3, ADCY5)^34^, ^35^ in DMD cardiomyocytes. Briefly, G‐protein mediated activation of AC, increases cAMP, activating protein kinase A (PKA) which in turn phosphorylates several targets, such as phospholamban, LTCC and Troponin I.^36^, ^37^

### 3.6 Western blot analysis of ATP2ase2 SERCA2 in DMD iPSC‐CMs and healthy iPSC‐CMs

To further understand the molecular mechanisms underlying the depressed inotropic response of DMD cardiomyocytes and the depleted SR Ca^2+^ stores, we performed Western blot analysis of ATPase 2 SERCA2 in healthy and DMD iPSC‐CMs (Figures S3 and S4). As shown in Figures S3 and S4, compared to healthy, SERCA2 is overexpressed in adult male and adult female cardiomyocytes. Based on this finding, we suggest that impaired [Ca^2+^]_i_ handling may be compensated by overexpression of SERCA2 in DMD iPSC‐CMs.

## 4 DISCUSSION

To decipher the mechanisms underlying the depressed ventricular function in DMD patients we tested the hypothesis that DMD iPSC‐CMs feature blunted positive inotropic response to β‐adrenergic stimulation. The major findings were: (a) blunted positive inotropic response to isoproterenol and elevated [Ca^2+^]_o_; (b) healthy‐like positive chronotropic response in DMD cardiomyocytes; (c) depressed SR Ca^2+^ release in DMD cardiomyocytes, likely to underlie the attenuated positive inotropic response; (d) RNA‐expression levels of specific subunits of the L‐type calcium channel, β1‐adrenergic receptor (ADRβ1) and adenylate cyclase are down‐regulated in DMD cardiomyocytes, which collectively may contribute to the depressed β‐adrenergic inotropic responsiveness.

### 4.1 The β‐adrenergic positive inotropic response is blunted in DMD cardiomyocytes

Since a clinical hallmark of DMD cardiomyopathy is depressed ventricular function,^9^, ^10^, ^11^, ^12^, ^13^, ^14^, ^15^ we attempted to determine the cellular basis of this major pathology. Indeed, we found that male and female DMD cardiomyocytes exhibit a blunted positive inotropic response to β‐adrenergic stimulation (Figure 1) which can account for the depressed cardiac function in DMD patient.^1^, ^3^, ^4^, ^38^ To decipher the mechanisms underlying the blunted response, we tested whether the following elements are impaired in DMD cardiomyocytes: (a) β1‐adrenergic signalling cascade; (b) the [Ca^2+^]_i_‐handling and contractile machineries.

#### 4.1.1 Is the β‐adrenergic receptor impaired in DMD cardiomyocytes?

A depressed β‐adrenergic inotropic response can result from aberrant function and/or decreased receptor density in DMD cardiomyocytes. This option was negated by the electrophysiological results showing comparable and increased response of DMD adult female and adult male cardiomyocytes, respectively, to isoproterenol, compared to healthy cells (Figure 2). Collectively, these findings suggest that in DMD cardiomyocytes the β‐adrenergic receptor is functional, and even hyperactive (in male cardiomyocytes), which may contribute to the higher heart rate in DMD patient.^24^, ^38^

#### 4.1.2 Are the intracellular [Ca^2+^]_i_‐handling and contractile machineries depressed in DMD cardiomyocytes?

To determine whether the blunted inotropic response is due to depressed [Ca^2+^]_i_ and contractile machineries in DMD cardiomyocytes, we compared the [Ca^2+^]_i_ transient and contraction parameters in DMD and healthy cells. Since these parameters were comparable in DMD and healthy cardiomyocytes (Figure S2), we concluded that the [Ca^2+^]_i_ and contractile machineries are similar in healthy and DMD cardiomyocytes, and thus cannot account for the depressed inotropic response. The similarity in contraction parameters among healthy and DMD cardiomyocytes, despite the dystrophin mutations, can be explained by the experimental conditions in which the cardiomyocytes are unloaded, and thus not subjected to mechanical stress which is expected to cause depressed contractile function in DMD cardiomyocytes.

#### 4.1.3 Are DMD cardiomyocytes responsive to elevated [Ca^2+^]_o_?

Next, we investigated whether a downstream element mediating other/any positive inotropic intervention is impaired, by measuring the response to elevated [Ca^2+^]_o_; this intervention augments the L‐type Ca^2+^ current (I_Ca,L_), which in turn increases SR Ca^2+^ release thereby increasing contractile force.^21^ These experiments show (Figure 3) that the inotropic response of DMD cardiomyocytes to elevated [Ca^2+^]_o_ is blunted (similar to that of isoproterenol) compared to healthy cardiomyocytes, suggesting that a key downstream element(s) mediating positive inotropic responses is compromised in DMD cells.

#### 4.1.4 Are SR Ca^2+^‐stores depleted in DMD cardiomyocytes?

The findings regarding the blunted inotropic response of DMD cardiomyocytes to isoproterenol and [Ca^2+^]_o_ lead us to hypothesize that the SR‐Ca^2+^‐handling capacity is impaired. The first step in testing this hypothesis was to measure the response to caffeine, which induces SR‐Ca^2+^ release by reducing the activation threshold of RyR2.^29^ In agreement with our previous studies,^21^, ^22^, ^29^ in healthy cardiomyocytes caffeine induced a long‐lasting rise in [Ca^2+^]_i_ along with a transient depression of the contractions, followed by a gradual resumption of contractions. In contrast, DMD cardiomyocytes exhibited a much shorter response to caffeine (Figure 4B‐D). In addition, the recovery time of DMD cardiomyocytes was shorter (Figure 4D), the area and amplitude were smaller (Figure 4B‐C), collectively suggesting that indeed in DMD cardiomyocytes SR‐Ca^2+^ stores are depleted, thereby underlying the blunted positive inotropic response to increased isoproterenol and elevated [Ca^2+^]_o_. To further support the notion of defective SR function in DMD cardiomyocytes, we tested the effect of ryanodine which blocks Ca^2+^‐induced SR‐Ca^2+^ release. In agreement with studies in iPSC‐CMs, hESC‐CMs^39^, ^40^, ^41^ and rabbit cardiomyocytes,^42^ in healthy cardiomyocytes ryanodine (30 μmol/L) attenuated all contraction parameters. As predicted from Ca^2+^‐depleted SR stores, in DMD cardiomyocytes the negative inotropic response was smaller than in healthy cardiomyocytes (Figure 5). To strengthen the hypothesis of depleted SR Ca^2+^ stores in DMD iPSC‐CMs, we compared the negative inotropic effect of the SERCA2 inhibitor CPA, in healthy and DMD iPSC‐CMs. Like ryanodine, in DMD cardiomyocytes CPA caused a smaller inotropic effect than in healthy cardiomyocytes (Figure 5E‐G), suggesting that SERCA2 function in DMD differs from healthy cardiomyocytes. Further, the overexpression of SERCA in DMD cardiomyocytes can account for a small negative inotropic effect of CPA in DMD cardiomyocytes.

The results from the caffeine, ryanodine and CPA experiments suggest that in DMD cardiomyocytes, the depressed positive inotropic response to isoproterenol and elevated [Ca^2+^]_o_ result in part from depleted SR Ca^2+^ stores; a most likely cause underlying this phenomenon is leaky RyR2. In this regards, Fauconnier et al^17^, ^43^ reported that structural and functional remodelling of the cardiac SR Ca^2+^ release channel/ryanodine receptor (RyR2) occurs in cardiomyocytes from *mdx* mice. RyR2 from *mdx* hearts were S‐nitrosylated and depleted of calstabin2 (FKBP12.6), resulting in ‘leaky’ RyR2 channels and a diastolic SR Ca^2+^ leak. This report on ‘leaky’ RyR2 channels from *mdx* hearts supports our proposed mechanism that in DMD cardiomyocytes the SR is Ca^2+^ depleted.

#### 4.1.5 The molecular mechanisms underlying the blunted positive inotropic response in DMD cardiomyocytes

To decipher the molecular mechanisms contributing to the blunted positive inotropic response in DMD cardiomyocytes, we performed a comprehensive RNA‐seq analysis. Importantly, we found that the expression of the β1‐adrenergic receptor (ADRβ1), predominantly expressed in the heart^32^, ^44^, ^45^ was down‐regulated by 2.8‐fold in DMD compared to healthy cardiomyocytes. The next step downstream to ADRβ1 is activation of adenylyl cyclase (AC) via Gs, resulting in increased cAMP levels. The primary target of cAMP is protein kinase A (PKA) which phosphorylates several key proteins essential for cardiac function, such as the L‐type calcium channel, phospholamban, troponin I and RyR.^46^ Therefore, because ADRβ1 is down‐regulated, its stimulation will result in attenuated AC activation, decreased cAMP levels, and consequently blunted positive inotropic response to isoproterenol. Our finding that ADRβ1 expression is decreased in DMD cardiomyocytes is supported by Li et al^47^ who showed that the abundance of ADRβ1 protein is reduced by 2/3 in 4‐month‐old *mdx* hearts compared to control hearts. Additionally, Lu et al examined β1‐adrenergic receptors from both left and right atria in young (12 week) and old mdx male mice (12 month), compared with their age‐ and sex‐matched healthy controls (C57). Briefly, mdx mice exhibited decreased response to isoprenaline compared to control mice.^48^ In addition, young and old *mdx* mice expressed a reduction in calcium‐induced positive inotropy compared to control mice, implying calcium handling abnormalities in *mdx* cardiac muscle. Overall, the complex alterations in β1‐adrenergic mechanisms and the dysfunction of calcium homeostasis may both contribute to the abnormalities of contractile function in *mdx* myocardium.^48^

The comprehensive RNA‐seq analysis further show that the AC III and V isoforms are down‐regulated by 3‐fold in DMD cardiomyocytes. Specifically, AC V which is the major cardiac isoform regulates heart rate and contractility.^35^ Therefore, down‐regulation of AC, especially type V, contributes to reduced cAMP levels and to depressed β‐adrenergic responsiveness. Another important element involved in the β‐adrenergic inotropic responsiveness is the L‐type Ca^2+^channel responsible for Ca^2+^ entry during the plateau of the action potential, thereby initiating contraction.^49^ The RNA‐seq analysis show a 3.5‐fold down‐regulation of the genes CACNA2D2^50^ and CACNG6 encoding for alpha‐2/delta and gamma subunits,^51^ respectively of the L‐type Ca^2+^channel. This finding is supported by Viola et al^49^ who demonstrated that Ca^2+^ influx is increased in *mdx* cardiomyocytes, as a consequence of delayed inactivation of I_Ca,L_, thereby contributing to increased cytoplasmic Ca^2+^. Moreover, activation of I_Ca,L_ was shown to increase mitochondrial Ca^2+^, NADH and superoxide concentrtions, as a result of increased uptake of Ca^2+^ into the mitochondria. Therefore, the L‐type Ca^2+^ channel contributes to impairments in mitochondrial Ca^2+^ handling in *mdx* ventricular cardiomyocytes. Although RNA levels of the L‐type Ca^2+^ channel were down‐regulated, the [Ca^2+^]_i_ transient properties in DMD cardiomyocytes did not differ from healthy cells. This may imply that the decrease in CACNA2D2 and CACNG6 expression was not significant enough to cause functional changes at a basal resting state of the channel. However, under stress conditions such as β‐adrenergic stimulation, the lack of sufficient channel expression manifested in DMD iPSC‐CMs contributes to the blunted β‐adrenergic inotropic responsiveness. Finally, MYL2 was found to be up‐regulated by‐2 fold in DMD cardiomyocytes. MYL2, known as the regulatory light chain of myosin, is a sarcomeric protein that plays a role in heart development and function. Following phosphorylation, MYL2 is involved in cross‐bridge cycling kinetics and cardiac muscle contraction.^52^, ^53^ This finding is consistent with Baker et al^54^ who demonstrated by means of RT‐PCR that MYL2 transcription is up‐regulated in mice lacking both dystrophin and utrophin as well as in *mdx* myotubes. The up‐regulation of MYL2 in *mdx* myotubes strengthens our finding in DMD cardiomyocytes.

#### 4.1.6 The SERCA data

To further decipher putative mechanisms underlying the depleted SR Ca^2+^ stores in DMD iPSC‐CMs, we found that phosphorylated SERCA2 is overexpressed by 11‐fold in adult male and by 2.75 in adult female compared to healthy cardiomyocytes (Figures S3 and S4). This finding is consistent with similar findings in *mdx* mice, which express increased levels of the slow Ca^2+^‐pump isoform (SERCA2a) (2‐fold) in skeletal muscle.^55^ In contrast to the protein expression findings, RNA‐seq analysis did not show SERCA overexpression, which may indicate that while the overall transcription levels are not changed, SERCA2 activity is increased, as demonstrated by the overexpression of SERCA2 which probably underwent posttranslational modifications. Increased SERCA activity was previously demonstrated to have a beneficial effect on failing hearts,^52^, ^53^, ^56^, ^57^, ^58^ and therefore, the increase SERCA expression can constitute a compensatory attempt to increase SR Ca^2+^‐stores and subsequently DMD iPSC‐CMs inotropy.

## 5 SUMMARY AND CONCLUSIONS

Our results demonstrate that DMD iPSC‐CMs exhibit blunted positive inotropic response to isoproterenol and elevated [Ca^2+^]_o_ compared to healthy cardiomyocytes. In addition, the results from the caffeine, ryanodine and CPA experiments suggest that in DMD cardiomyocytes these depressed responses result from depleted SR Ca^2+^ stores. We propose that SERCA2 overexpression in DMD cardiomyocytes constitutes a compensatory mechanism to increase SR Ca^2+^‐stores and subsequently DMD iPSC‐CMs inotropy. The RNA‐seq data demonstrate that down‐regulation of ADRβ1 and major AC isoforms contribute to the blunted positive inotropic response to isoproterenol. These results emphasize the involvement of abnormal β‐adrenergic cascade and [Ca^2+^]_i_‐handling in DMD cardiac pathophysiology and may provide the basis for further research and the development of new therapeutic agents targeting these abnormalities.

## CONFLICT OF INTEREST

The authors confirm that there are no conflicts of interest.

## AUTHOR CONTRIBUTIONS

**Lucy N. Mekies:** Data curation (lead); Formal analysis (lead); Investigation (lead); Methodology (lead); Writing‐original draft (lead); Writing‐review & editing (lead). **Danielle Regev:** Formal analysis (supporting); Methodology (equal). **Binyamin Eisen:** Data curation (supporting); Formal analysis (supporting); Writing‐review & editing (supporting); Methodology (equal). **Jonatan Fernandez‐Gracia:** Formal analysis (supporting); Methodology (equal). **Polina Baskin:** Writing‐review & editing (supporting); Methodology (equal). **Ronen Ben Jehuda:** Investigation (supporting); Methodology (supporting). **Rita Shulman:** Methodology (supporting). **Irina Reiter:** Methodology (supporting). **Raz Palty:** Conceptualization (supporting); Investigation (supporting). **Michael Arad:** Conceptualization (supporting); Investigation (supporting). **Eyal Gottlieb:** Conceptualization (supporting); Resources (supporting). **Ofer Binah:** Conceptualization (lead); Resources (lead); Supervision (lead); Validation (lead); Visualization (lead).

## Supporting information

Supplementary Material

## References

[1] Mavrogeni S , Markousis‐Mavrogenis G , Papavasiliou A , Kolovou G . Cardiac involvement in Duchenne and Becker muscular dystrophy. World J Cardiol. 2015;7:410‐414. 26225202 10.4330/wjc.v7.i7.410 PMC4513493

[2] Eagle M , Baudouin SV , Chandler C , et al. Survival in Duchenne muscular dystrophy: improvements in life expectancy since 1967 and the impact of home nuctural ventilation. Neuromuscul Disord. 2002;12:926‐929. 12467747 10.1016/s0960-8966(02)00140-2

[3] Bushby K , Finkel R , Birnkrant DJ , et al. Diagnosis and management of Duchenne muscular dystrophy, part 2: implementation of multidisciplinary care. Lancet Neurol. 2010;9:177‐189. 19945914 10.1016/S1474-4422(09)70272-8

[4] McNally EM , Kaltman JR , Benson DW , et al. Contemporary cardiac issues in Duchenne muscular dystrophy. Circulation. 2015;131:1590‐1598. 25940966 10.1161/CIRCULATIONAHA.114.015151 PMC4573596

[5] Gumerson JD , Michele DE . The dystrophin‐glycoprotein complex in the prevention of muscle damage. J Biomed Biotechnol. 2011;2011:1‐13. 10.1155/2011/210797 PMC3189583 22007139

[6] Bushby K , Finkel R , Birnkrant DJ , et al. Diagnosis and management of Duchenne muscular dystrophy, part 1: diagnosis, and pharmacological and psychosocial management. Lancet Neurol. 2010;9:77‐93. 19945913 10.1016/S1474-4422(09)70271-6

[7] Finsterer J , Stollberger C . The heart in human dystrophinopathies. Cardiology. 2003;99:1‐19. 10.1159/000068446 12589117

[8] Spurney CF . Cardiomyopathy of Duchenne muscular dystrophy: Current understanding and future directions. Muscle Nerve. 2011;44:8‐19. 21674516 10.1002/mus.22097

[9] Angelini C , Di LR , Cudia P . Autonomic regulation in muscular dystrophy. Front Physiol. 2013;4:257. 24065927 10.3389/fphys.2013.00257 PMC3778236

[10] Sabharwal R . Autonomic regulation in muscular dystrophy. Front Physiol. 2014;5:61. 24596560 10.3389/fphys.2014.00061 PMC3925827

[11] Sabharwal R . The link between stress disorders and autonomic dysfunction in muscular dystrophy. Front Physiol. 2014;5:25. 24523698 10.3389/fphys.2014.00025 PMC3905207

[12] da Silva TD , Massetti T , Crocetta TB , et al. Heart rate variability and cardiopulmonary dysfunction in patients with Duchenne muscular dystrophy: A systematic review. Pediatr Cardiol. 2018;39:869‐883. 29696428 10.1007/s00246-018-1881-0

[13] Alvarez MPB , da Silva TD , Favero FM , et al. Autonomic modulation in duchenne muscular dystrophy during a computer task: a prospective control trial. PLoS One. 2017;12(1):e0169633. 28118369 10.1371/journal.pone.0169633 PMC5261738

[14] Dittrich S , Tuerk M , Haaker G , et al. Cardiomyopathy in Duchenne muscular dystrophy: current value of clinical, electrophysiological and imaging findings in children and teenagers. Klin Pädiatrie. 2015;227:225‐231. 10.1055/s-0034-1398689 26058601

[15] Thomas TO , Jefferies JL , Lorts A , et al. Autonomic dysfunction: A driving force for myocardial fibrosis in young Duchenne muscular dystrophy patients? Pediatr Cardiol. 2015;36:561‐568. 25399404 10.1007/s00246-014-1050-z

[16] Eisen B , Ben JR , Cuttitta AJ , et al. Electrophysiological abnormalities in induced pluripotent stem cell‐derived cardiomyocytes generated from Duchenne muscular dystrophy patients. J Cell Mol Med. 2019;23:2125‐2135. 30618214 10.1111/jcmm.14124 PMC6378185

[17] Fauconnier J , Thireau J , Reiken S , et al. Leaky RyR2 trigger ventricular arrhythmias in Duchenne muscular dystrophy. Proc Natl Acad Sci. 2010;107:1559‐1564. 20080623 10.1073/pnas.0908540107 PMC2824377

[18] Garbincius JF , Michele DE . Dystrophin–glycoprotein complex regulates muscle nitric oxide production through mechanoregulation of AMPK signaling. Proc Natl Acad Sci. 2015;112:13663‐13668. 26483453 10.1073/pnas.1512991112 PMC4640723

[19] Bellinger AM , Reiken S , Carlson C , et al. Hypernitrosylated ryanodine receptor calcium release channels are leaky in dystrophic muscle. Nat Med. 2009;15:325‐330. 19198614 10.1038/nm.1916 PMC2910579

[20] Lian X , Zhang J , Azarin SM , et al. Directed cardiomyocyte differentiation from human pluripotent stem cells by modulating Wnt/β‐catenin signaling under fully defined conditions. Nat Protoc. 2013;8:162‐175. 23257984 10.1038/nprot.2012.150 PMC3612968

[21] Hallas T , Eisen B , Shemer Y , et al. Investigating the cardiac pathology of SCO2‐mediated hypertrophic cardiomyopathy using patients induced pluripotent stem cell–derived cardiomyocytes. J Cell Mol Med. 2018;22:913‐925. 29193756 10.1111/jcmm.13392 PMC5783844

[22] Schick R , Mekies LN , Shemer Y , et al. Functional abnormalities in induced pluripotent stem cell‐derived cardiomyocytes generated from titin‐mutated patients with dilated cardiomyopathy. PLoS One. 2018;13:1‐25. 10.1371/journal.pone.0205719 PMC6192629 30332462

[23] Ben‐Ari M , Naor S , Zeevi‐Levin N , et al. Developmental changes in electrophysiological characteristics of human‐induced pluripotent stem cell‐derived cardiomyocytes. Heart Rhythm. 2016;13:2379‐2387. 27639456 10.1016/j.hrthm.2016.08.045 PMC5421365

[24] Rajdev A , Fellow CE , Groh WJ . Arrhythmias in the muscular dystrophies. Card Electrophysiol Clin. 2015;7:303‐308. 26002394 10.1016/j.ccep.2015.03.011 PMC4441951

[25] Stoehr A , Neuber C , Baldauf C , et al. Automated analysis of contractile force and Ca^2+^ transients in engineered heart tissue. Am J Physiol Heart Circ Physiol. 2014;306:H1353‐H1363. 24585781 10.1152/ajpheart.00705.2013 PMC4116534

[26] Andersson DC , Meli AC , Reiken S , et al. Leaky ryanodine receptors in β‐sarcoglycan deficient mice: a potential common defect in muscular dystrophy. Skelet Muscle. 2012;2(1):9. 22640601 10.1186/2044-5040-2-9 PMC3605002

[27] Divet A , Lompre AM , Huchet‐Cadiou C . Effect of cyclopiazonic acid, an inhibitor of the sarcoplasmic reticulum Ca‐ATPase, on skeletal muscles from normal and mdx mice. Acta Physiol Scand. 2005;184:173‐186. 15954985 10.1111/j.1365-201X.2005.01450.x

[28] Sedan O , Dolnikov K , Zeevi‐Levin N , et al. 1,4,5‐inositol trisphosphate‐operated intracellular calcium stores and angiotensin‐II/endothelin‐1 signaling pathway are functional in human Embryonic Stem Cell‐derived cardiomyocytes. Stem Cells. 2008;26:3130‐3138. 18818435 10.1634/stemcells.2008-0777

[29] Novak A , Barad L , Lorber A , Gherghiceanu M , Rappaport B . Functional abnormalities in iPSC‐derived cardiomyocytes generated from CPVT1 and CPVT2 patients carrying ryanodine or calsequestrin mutations. J Cell Mol Med. 2015;19:2006‐2018. 26153920 10.1111/jcmm.12581 PMC4549051

[30] Mannhardt I , Eder A , Dumotier B , et al. Blinded contractility analysis in hipsc‐cardiomyocytes in engineered heart tissue format: comparison with human atrial trabeculae. Toxicol Sci. 2017;158:164‐175. 28453742 10.1093/toxsci/kfx081 PMC5837217

[31] Carlsson E , Dahlöf CG , Hedberg A , Persson H , Tångstrand B . Differentiation of cardiac chronotropic and inotropic effects of β‐adrenoceptor agonists. Naunyn Schmiedebergs Arch Pharmacol. 1977;300:101‐105. 22823 10.1007/BF00505039

[32] Lohse MJ , Engelhardt S , Eschenhagen T . What is the role of β‐adrenergic signaling in heart failure? Circ Res. 2003;93:896‐906. 14615493 10.1161/01.RES.0000102042.83024.CA

[33] Motiejunaite J , Amar L , Vidal‐Petiot E . Adrenergic receptors and cardiovascular effects of catecholamines. Ann Endocrinol (Paris). 2020. 10.1016/j.ando.2020.03.011. Online ahead of print. 32473788

[34] Susheela AK , Kaul RD , Sachdeva K , Singh N . Adenyl cyclase activity in Duchenne dystrophic muscle. J Neurol Sci. 1975;24:361‐363. 10.1016/0022-510x(75)90256-7 1117311

[35] Vatner SF , Park M , Yan L , et al. Adenylyl cyclase type 5 in cardiac disease, metabolism, and aging. Am J Physiol Heart Circ Physiol. 2013;305:1‐8. 10.1152/ajpheart.00080.2013 PMC3727099 23624627

[36] Sadana R , Dessauer CW . Physiological roles for G protein‐regulated adenylyl cyclase isoforms: Insights from knockout and overexpression studies. Neurosignals. 2009;17:5‐22. 18948702 10.1159/000166277 PMC2790773

[37] Zaccolo M . CAMP signal transduction in the heart: Understanding spatial control for the development of novel therapeutic strategies. Br J Pharmacol. 2009;158:50‐60. 19371331 10.1111/j.1476-5381.2009.00185.x PMC2795260

[38] Dhargave P , Nalini A , Abhishekh HA , et al. Assessment of cardiac autonomic function in patients with Duchenne muscular dystrophy using short term heart rate variability measures. Eur J Paediatr Neurol. 2014;18:317‐320. 24445161 10.1016/j.ejpn.2013.12.009

[39] Lee YK , Ng KM , Lai WH , et al. Calcium homeostasis in human Induced Pluripotent Stem Cell‐derived cardiomyocytes. Stem Cell Rev Reports. 2011;7:976‐986. 10.1007/s12015-011-9273-3 PMC3226695 21614516

[40] Itzhaki I , Rapoport S , Huber I , et al. Calcium handling in human Induced Pluripotent Stem Cell derived cardiomyocytes. PLoS One. 2011;6:e18037. 21483779 10.1371/journal.pone.0018037 PMC3069979

[41] Germanguz I , Sedan O , Zeevi‐Levin N , et al. Molecular characterization and functional properties of cardiomyocytes derived from human inducible pluripotent stem cells. J Cell Mol Med. 2011;15:38‐51. 20041972 10.1111/j.1582-4934.2009.00996.x PMC3822492

[42] Satoh H . Electrophysiological actions of ryanodine on single rabbit sinoatrial nodal cells. Gen Pharmacol. 1997;28:31‐38. 9112074 10.1016/s0306-3623(96)00182-6

[43] Lehnart S , Marks AR . Regulation of ryanodine receptors in the heart. Circ Res. 2007;101:746‐749. 17932330 10.1161/CIRCRESAHA.107.162479

[44] Grinshpon M , Bondarenko VE . Simulation of the effects of moderate stimulation/inhibition of the β1‐adrenergic signaling system and its components in mouse ventricular myocytes. Am J Physiol Cell Physiol. 2016;310:C844‐C856. 26936457 10.1152/ajpcell.00002.2016

[45] Hammer KP , Ljubojevic S , Ripplinger CM , Pieske BM , Bers DM . Cardiac myocyte alternans in intact heart: Influence of cell–cell coupling and β‐adrenergic stimulation. J Mol Cell Cardiol. 2015;84:1‐9. 25828762 10.1016/j.yjmcc.2015.03.012 PMC4500534

[46] Saad NS , Elnakish MT , Ahmed AAE , Janssen PML . Protein kinase A as a promising target for heart failure drug development. Arch Med Res. 2018;49:530‐537. 30642654 10.1016/j.arcmed.2018.12.008 PMC6451668

[47] Li Y , Wu G , Tang Q , et al. Slow cardiac myosin regulatory light chain 2 (MYL2) was down‐expressed in chronic heart failure patients. Clin Cardiol. 2011;34:30‐34. 21259275 10.1002/clc.20832 PMC6652384

[48] Lu S , Hoey A . Age‐ and sex‐associated changes in cardiac β1‐adrenoceptors from the muscular dystrophy (mdx) mouse. J Mol Cell Cardiol. 2000;32:1661‐1668. 10966828 10.1006/jmcc.2000.1200

[49] Viola HM , Davies SMK , Filipovska A , Hool LC . L‐type calcium channel contributes to alterations in mitochondrial calcium handling in the mdx ventricular myocyte. Am J Physiol Heart Circ Physiol. 2013;304:767‐775. 10.1152/ajpheart.00700.2012 23335798

[50] Gao B , Sekido Y , Maximov A , et al. Functional properties of a new voltage‐dependent calcium channel auxiliary subunit gene (CACNA2D2). J Biol Chem. 2000;275:12237‐12242. 10766861 10.1074/jbc.275.16.12237 PMC3484885

[51] Hofmann F , Flockerzi V , Kahl S , Wegener JW . L‐type CaV1.2 calcium channels: From in vitro findings to in vivo function. Physiol Rev. 2014;94:303‐326. 24382889 10.1152/physrev.00016.2013

[52] Ahlers BA , Song J , Wang JF , et al. Effects of sarcoplasmic reticulum Ca2+‐ATPase overexpression in postinfarction rat myocytes. J Appl Physiol. 2005;98:2169‐2176. 15677742 10.1152/japplphysiol.00013.2005

[53] Isner JM . Myocardial gene therapy. Nature. 2002;415:234‐239. 11805848 10.1038/415234a

[54] Baker PE , Kearney JA , Gong B , et al. Analysis of gene expression differences between utrophin/dystrophin‐ deficient vs mdx skeletal muscles reveals a specific upregulation of slow muscle genes in limb muscles. Neurogenetics. 2006;7:81‐91. 16525850 10.1007/s10048-006-0031-7

[55] Scharf M , Neef S , Freund R , et al. Mitogen‐activated protein kinase‐activated protein kinases 2 and 3 regulate SERCA2a expression and fiber type composition to modulate skeletal muscle and cardiomyocyte function. Mol Cell Biol. 2013;33:2586‐2602. 23608535 10.1128/MCB.01692-12 PMC3700115

[56] Inesi G , Prasad AM , Pilankatta R . The Ca^2+^ ATPase of cardiac sarcoplasmic reticulum: physiological role and relevance to diseases. Biochem Biophys Res Commun. 2008;369:182‐187. 18068669 10.1016/j.bbrc.2007.11.161 PMC2323400

[57] Lipskaia L , Chemaly ER , Hadri L , Lompre AM , Hajjar RJ . Sarcoplasmic reticulum Ca2 ATPase as a therapeutic target for heart failure. Expert Opin Biol Ther. 2010;10:29‐41. 20078230 10.1517/14712590903321462 PMC3001226

[58] Goonasekera SA , Kranias EG , Molkentin JD , et al. Mitigation of muscular dystrophy in mice by SERCA overexpression in skeletal muscle Find the latest version: mitigation of muscular dystrophy in mice by SERCA overexpression in skeletal muscle. J Clin Invest. 2011;121:1044‐1052. 21285509 10.1172/JCI43844 PMC3049367

