# Supplementary material for: Depressed β‐adrenergic inotropic responsiveness and intracellular calcium handling abnormalities in Duchenne Muscular Dystrophy patients’ induced pluripotent stem cell–derived cardiomyocytes
Source: J Cell Mol Med. 2021 Feb 22;25(8):3922–34. doi: 10.1111/jcmm.16341 (PMC8051742; doi:10.1111/jcmm.16341)
Supplement: Supplementary file 1 — Supplementary Material [file JCMM-25-3922-s001.docx]

**Depressed β-adrenergic inotropic responsiveness and intracellular calcium handling abnormalities in Duchenne muscular dystrophy patients’ induced pluripotent stem cell-derived cardiomyocytes**

^1^Lucy N Mekies, ^1,*^Danielle Regev, ^1,*^Binyamin Eisen,^1^ ^2,*^Jonatan Fernandez-Gracia, ^1,*^Polina Baskin, ^1,3^Ronen Ben Jehuda, ^1^Rita Shulman, ^1^Irina Reiter, ^4^Raz Palty, ^5,6^Michael Arad, ^2^Eyal Gottlieb, ^1^Ofer Binah

^1^Department of Physiology, Biophysics and Systems Biology, Rappaport Faculty of Medicine, Technion – Israel Institute of Technology, Haifa, Israel. ^2^Department of Cell Biology and Cancer Science, Rappaport Faculty of Medicine, Technion – Israel Institute of Technology, Haifa, Israel. ^3^Faculty of Biotechnology and Food Engineering, Technion – Israel Institute of Technology, Haifa, Israel. ^4^Department of Biochemistry, Rappaport Faculty of Medicine, Technion – Israel Institute of Technology, Haifa, Israel. ^5^Leviev Heart Center, Sheba Medical Center, Ramat Gan, Israel. ^6^Sackler Faculty of Medicine, Tel Aviv University, Tel Aviv, Israel.

**^*^These authors contributed equally to the manuscript.**

Corresponding author

Ofer Binah, PhD

Department of Physiology, Biophysics and System Biology

Rappaport Faculty of Medicine, Technion
POB 9649, Haifa, 31096 Israel

Email: [binah@tx.technion.ac.il](mailto:binah@tx.technion.ac.il); Tel: +972-4-8295262

**Detailed Methods**

Measurements of intracellular Ca^2+^ transients and contractions

Intracellular Ca^2+^ ([Ca^2+^]_i_) transients and contractions were recorded from small contracting 45-65-day old embryoid bodies (EBs) by means of fura-2 fluorescence and video edge detector, respectively, using the IonOptix Calcium and Contractility system (Westwood, MA, USA).^1–3^ In brief, spontaneously contracting EBs were mechanically dissected and adhered onto 18 mm diameter gelatin-coated glass slides. Subsequently, fura-2-stained (2.5 μM) contracting areas were transferred to a chamber mounted on the stage of an inverted microscope and perfused at a rate of 1–1.5 ml/min Tyrode’s solution at 37^0^C. The Tyrode’s solution contains (mmol/L): 140 NaCl, 5.4 KCl, 1 MgCl_2_, 2 sodium pyrovate, 1 CaCl_2_, 10 HEPES, 10 glucose (pH 7.4 adjusted with NaOH). The EBs were paced at 0.5-2.5 Hz which corresponded to a frequency 20-50% higher than the spontaneous beating rate. The acquisition rate of both the [Ca^2+^]_i_ transients and contractions was 100 points/sec. Analysis was performed by averaging 20 consecutive signals using the IonOptix designated system. To characterize the [Ca^2+^]_i_ transients the following prameters were calculated: Amplitude (R_Amp_) - the differences between maximal (‘systolic’) and minimal (‘diastolic’) ratio values. The maximal rates of [Ca^2+^]_i_ rise (+d[Ca^2+^]_i_/dt) and decay (-d[Ca^2+^]_i_/dt). To characterize the contractions the following parameters were calculated: Amplitude (L_Amp_) - the difference between minimal and maximal video cursor positions. The maximal rates of contraction (+dL/dt) and relaxation (-dL/dt).

Action potential recordings

For action potential recordings, spontaneously contracting areas of monolayer culture were mechanically/enzymatically (0.25% trypsin-EDTA, Biological Industries, Beit-Haemek, Israel) dissociated and dispersed. This dispersion resulted in single cell-to-small clusters, which were plated on Matrigel-coated glass coverslips (13 mm diameter) in 24-well plates supplemented with 10 μM blebbistatin. Coverslips were incubated at 37°C for 2-4 days, followed by a 5 day recovery period without blebbistatin before performing electrophysiological experiments.^4^ In all experiments, the coverslips were perfused at 37°C with an external solution containing (in mmol/L): 140 NaCl, 5.4 KCl, 1.8 CaCl_2_, 1 MgCl_2_, 10 glucose and 10 HEPES titrated to pH 7.4 with NaOH (310 mOsm). The patch pipette solution contained (mmol/L): 120 KCl, 1 MgCl_2_, 3 Mg-ATP, 10 HEPES, and 10 EGTA titrated to pH 7.2 with KOH and adjusted at 290 mOsm with saccharose (all materials were purchased from Sigma-Aldrich, Rehovot, Israel). Action potentials were recorded from cardiomyocyte clusters. Axopatch 200B, Digidata 1322 or 1440 and pClamp10 (Molecular Devices, Sunnyvale, CA, USA) were used for data amplification, acquisition and analysis. Signals were digitized at 4-10 kHz. Patch electrodes with resistances of 4-7 MΩ were pulled from borosilicate glass capillaries (Harvard Apparatus, Holliston, MA, USA). The recordings were analyzed for the detection of all peaks of the recorded signal from which action potential parameters and IBIs were calculated by using the dedicated MATLAB software.^4^

Western Blot

Cardiomyocytes were lysed in ice-cold lysis buffer containing RIPA buffer, anti-phosphatase (1:10), protease inhibitor (1:25). The suspension was centrifuge at 1,500 rpm for 10 min at 4°C and the supernatant aspirated. The pellet was resuspend in cold PBS, transferred to a TPX tube and centrifuged at 1,500 rpm for 10 min at 4°C, followed by aspiration of the supernatant. This process was repeated 2X. The extracts were then centrifuged at 20,000×g for 20 min at 4°C, and protein concentration was determined from diluted aliquots of the soluble fraction by BCA protein assay (Thermo Fisher Scientific, Waltham, MA, US). Subsequently, the samples were diluted in fresh lysis buffer to yield equivalent final protein concentrations. Lysates were mixed with lithium dodecyl sulfate sample buffer with DTT (50 mM) (nuPAGE, Invitrogen) and boiled at 95 °C for 5 min. Loading controls were run on the same blot. SDS-PAGE was conducted on pre-cast polyacrylamide gels (Nupage 4–12% Bis Tris gel, Novex, Invitrogen, Waltham, MA, USA) and transfered onto polyvinylidene difluoride membranes (Immun-Blot, Bio-Rad) using an electrophoretic transfer cell (Mini Trans-Blot, Bio-Rad Laboratories, Hercules, CA, US). Blocked membranes (5% milk/tris-buffered saline with Tween-20, TBST) were incubated with primary antibody overnight at 4⁰C, followed by TBST washes and secondary horseradish peroxidase (HRP)-conjugated antibody detection. Bands were visualized using ECL reagents (GE Healthcare, Buckinghamshire, UK), using ImageQuant LAS 4000. The following antibodies were used: anti-SERCA2 antibody (#4388) at 1:1,000 working concentration (MW 114 and 140 kDa), β-Tubulin Mouse mAb (D3U1W) at 1:1000 (MW 55 kDa) from Cell Signaling (New England BioLabs, Hertfordshire, UK); HRP-conjugated secondary antibodies used were anti-rabbit IgG and anti-mouse IgG from Jackson (West Grove, PA, USA).

RNA extraction

RNA extraction from 10^6^ cardiomyocytes was carried out using the “ReliaPrep™ RNA Cell Miniprep System Kit” (Promega, Fitchburg, WI, USA) according to the manufacturer’s instructions. First, cDNAs were enriched in genes involved in cardiovascular disease using a custom capture probe (Agilent, Santa Clara, CA, USA) library designed against the coding region. Extracted RNAs were processed using the “SureSelect Strand-Specific RNA Library Prep for Illumina Multiplexed Sequencing mRNA Library Preparation Protocol” (Agilent, Santa Clara, CA, USA) with modifications. Briefly, RNAs were processed up to the amplification and indexing step following the instructions. From this point, hybridization and post-hybridization steps were carried out using the “Sure Select XT target enrichment system for Ilumina Paired end sequencing library protocol” (Agilent, Santa Clara, CA, USA). The resulting libraries were sequenced on a 1500 Illumina Hi-Seq at 2 x 100 (Ilumina, San Diego, CA, USA). Scientific, Austin, TX, USA), following the manufacturer’s instructions. The resulting libraries were sequenced on a 1500 Illumina Hi-Seq at 2 x 100.

RNA-seq analysis

RNA-seq libraries were produced according to manufacture protocol (NEBNext Ultrall directional RNA library prep kit for Illumina, cat. no. E7760, (San Diego, CA, USA) using 800 ng total RNA. mRNAs pull-down was performed using Magnetic Isolation Module (NEB, cat. no. E7490). All libraries were mixed into a single tube with equal molarity. The RNA-seq data was generated on Illumina NextSeq500, 75 single-end read in high-output mode (Illumina, cat. no. 20024906). Quality control for the transcripts was assessed using Fastqc (v. 0.11.5), reads were trimmed for adapters, low quality 3’-ends and a minimum length of 20bp using CUTADAPT (v. 1.12). 83 bp reads were aligned using STAR ^5^ (v. 2.6.0a) to the Human reference genome (GRCh38.p13, downloaded from Ensembl with its corresponding GTF annotation file). The number of gene reads count matrix was generated using Rsubread ^6^(v.2.2.4) in R (v. 4.0.2). A total of 58395 transcripts were mapped to the genome. A count filter (at least 5 counts in at least 2 samples) was applied to discard 35426 low-count transcripts before differential expression analysis. Differential expression analysis between the DMD and the control group was carried out using the DESeq2 package ^7^ (v. 1.28.1) using default parameters of lfcThreshold of 0 and alpha of 0.05. Transcripts were annotated with the Ensembl using biomaRt ^8^ (v. 2.44.1). KEGG genesets were downloaded using the EnrichmentBrowser ^9^ package (v. 2.18.0).

Statistical analysis

Results are presented as mean±SEM. Comparisons between adult male, adult female and healthy iPSC-CMs were performed using One-Way or Two-Way ANOVA followed by Holm-Sidak test using SigmaPlot 12.0 software (Systat Software International, San Jose, CA, USA). A value of P<0.05 was considered statistically significant.

On-line Supplement Figure 1


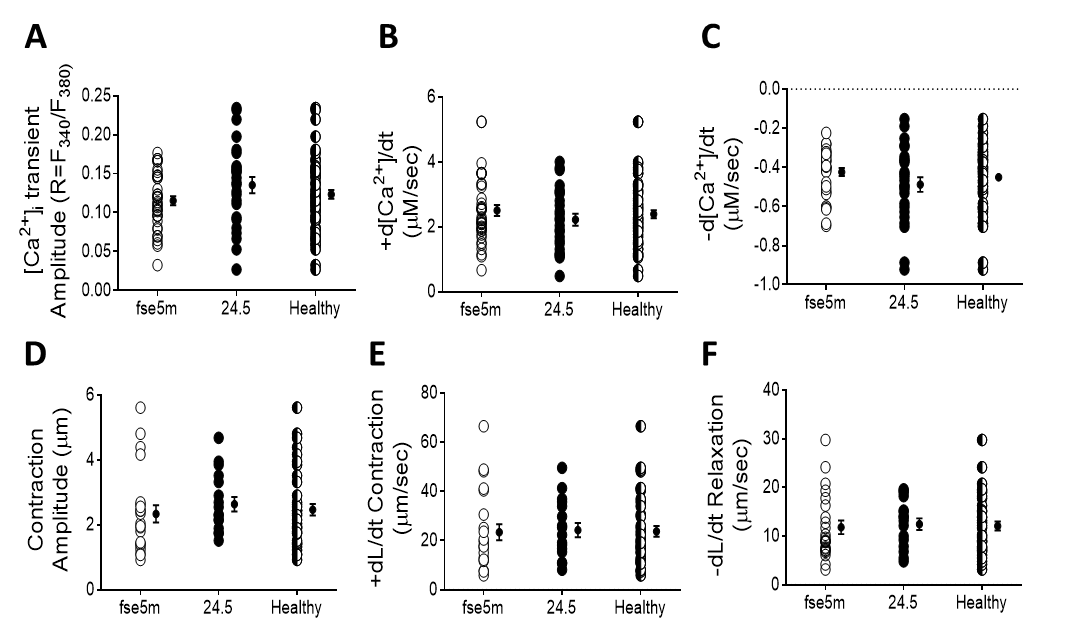


Figure 1

The [Ca^2+^]_i_ transient and contraction parameters in iPSC-CMs generated from 2 healthy volunteers; clones fse5m and 24.5. (A-C) [Ca^2+^]_i_ transient (clone fse5m, n=37; clone 24.5, n=26) amplitude and maximal rates of [Ca^2+^]_i_ rise and decay. (D-F) Contraction healthy (clone fse5m, n=23, clone 24.5, n=17) amplitude and maximal rates of contraction and relaxation. Next to each column of individual values, the Mean+SEM (filled symbol) is shown. One-way ANOVA was performed followed by Holm-Sidak test, **P* < 0.05, ***P* < 0.01 and ****P* < 0.001 vs healthy.

On-line Supplement Figure 2


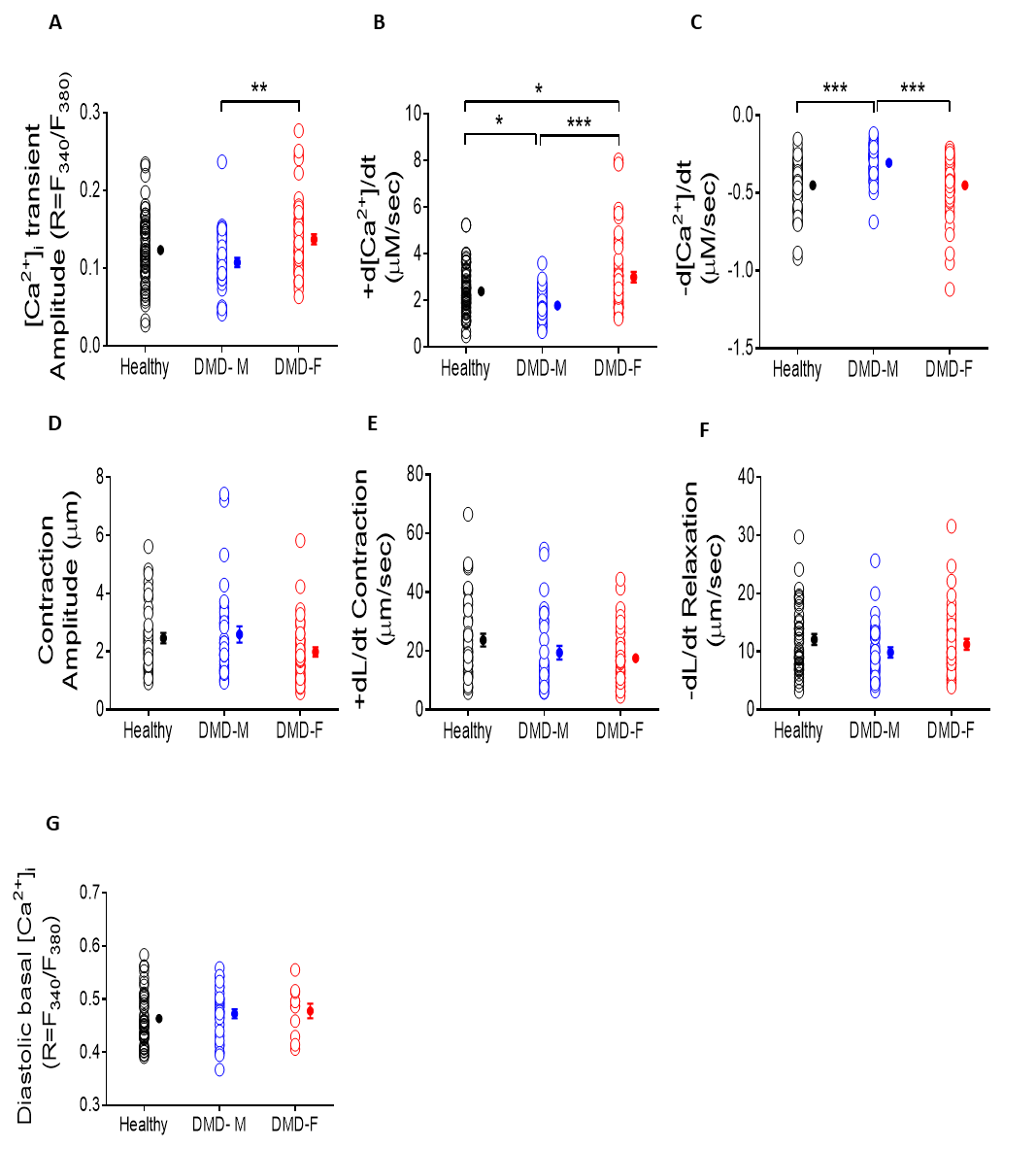


Figure 2

The [Ca^2+^]_i_ transient and contraction parametersin healthy and mutated (DMD adult male and adult female) iPSC-CM. (A-C) [Ca^2+^]_i_ transient healthy (fse5m clone n=37, 24.5 clone n=26; n=63), adult male (n=42), adult female (n=53) amplitude and maximal rates of [Ca^2+^]_i_ rise and decay, respectively. (D-F) Contraction healthy (fse5m clone n=23, 24.5 clone n=17; n=40), adult male (n=32), adult female (n=40) amplitude and maximal rates of contraction and relaxation, respectively. (G) Diastolic basal [Ca^2+]^i healthy (fse5m clone n=20 , 24.5 clone n=26 ; n=46 ), adult male (n=31), adult female (n=11 ). Next to each column of individual values, the Mean+SEM (filled symbol) is shown. One-way ANOVA was performed followed by Holm-Sidak test, **P* < 0.05, ***P* < 0.01 and *** *P* < 0.001 vs Healthy (clones fse5m + 24.5).

On-line Supplement Figure 3


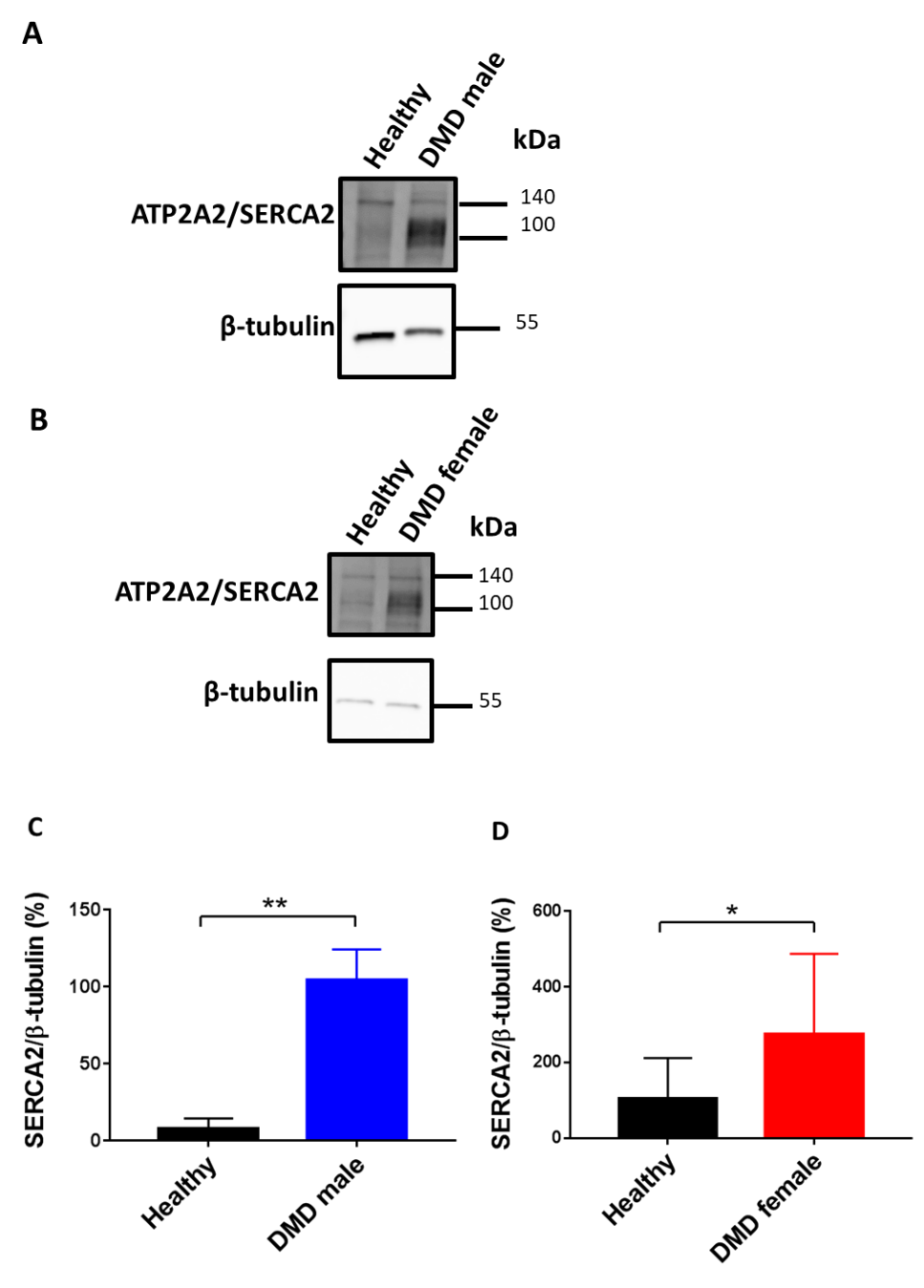


Figure 3

Western blot of healthy (A, B), DMD adult male (n=3) (A) and DMD adult female (n=3) (B). Quantification of ATPase 2 SERCA2 in DMD iPSC-CMs (male and female) and healthy iPSC-CMs normalized to β-tubulin (C, D). Uncropped Western blots are shown in Supplement Fig. 4. **P* < 0.05, ***P* < 0.01 vs Healthy (clones fse5m plus 24.5). Two-Sample t-test was performed.

On-line Supplement Figure 4


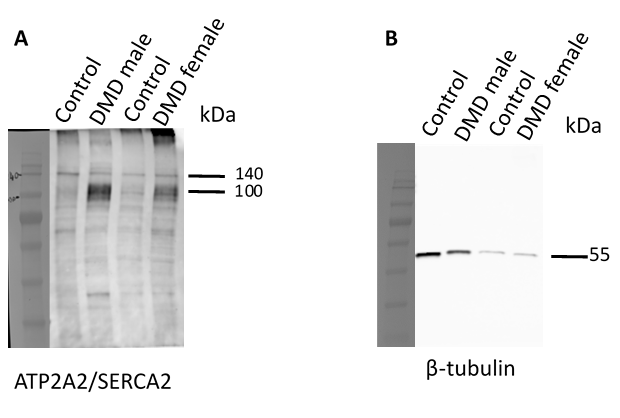


Figure 4

Uncropped Western blot of control, DMD adult male and DMD adult female (n=3) with ATP2A2/SERCA2 (A) and β-tubulin (B). A sharp demarcation between the molecular marker (ladder) and the control lanes results from the fact that the ladder was photographed with exposure conditions different from those used for the other lanes.

**References**

1. Dolnikov K, Shilkrut M, Zeevi-Levin N, Gerecht-Nir S, Amit M, Danon A, Itskovitz-Eldor J, Binah O. Functional properties of human embryonic stem cell-derived cardiomyocytes: intracellular Ca^2+^ handling and the role of sarcoplasmic reticulum in the contraction. *Stem Cells* 2006;**24**:236–245.

2. Sedan O, Dolnikov K, Zeevi-Levin N, Leibovich N, Amit M, Itskovitz-Eldor J, Binah O. 1,4,5-Inositol Trisphosphate-operated intracellular Ca^2+^ stores and angiotensin-II/endothelin-1 signaling pathways are functional in human embryonic stem cell-derived cardiomyocytes. *Stem Cells* 2008;**26**:3130–3138.

3. Novak A, Barad L, Zeevi-Levin N, Shick R, Shtrichman R, Lorber A, Itskovitz-Eldor J, Binah O. Cardiomyocytes generated from CPVTD307H patients are arrhythmogenic in response to β-adrenergic stimulation. *J Cell Mol Med* 2012;**16**:468–482.

4. Ben-Ari M, Schick R, Barad L, Novak A, Ben-Ari E, Lorber A, Itskovitz-Eldor J, Rosen MR, Weissman A, Binah O. From beat rate variability in induced pluripotent stem cell-derived pacemaker cells to heart rate variability in human subjects. *Heart Rhythm* 2014;**11**:1808–1818.

5. Dobin A, Davis CA, Schlesinger F, Drenkow J, Zaleski C, Jha S, Batut P, Chaisson M, Gingeras TR. STAR: Ultrafast universal RNA-seq aligner. *Bioinformatics* 2013;**29**:15–21.

6. Liao Y, Smyth GK, Shi W. The R package Rsubread is easier, faster, cheaper and better for alignment and quantification of RNA sequencing reads. *Nucleic Acids Res* 2019;**47**.

7. Love MI, Huber W, Anders S. Moderated estimation of fold change and dispersion for RNA-seq data with DESeq2. *Genome Biol* 2014;**15**:1–21.

8. Smedley D, Haider S, Ballester B, Holland R, London D, Thorisson G, Kasprzyk A. BioMart - Biological queries made easy. *BMC Genomics* 2009;**10**:1–12.

9. Geistlinger L, Csaba G, Zimmer R. Bioconductor’s EnrichmentBrowser: Seamless navigation through combined results of set- & network-based enrichment analysis. *BMC Bioinformatics* 2016;**17**:1–11.
